# Gonadotropin and steroid hormones regulate pluripotent very small embryonic-like stem cells in adult mouse uterine endometrium

**DOI:** 10.1186/s13048-018-0454-4

**Published:** 2018-09-21

**Authors:** Kreema James, Deepa Bhartiya, Ranita Ganguly, Ankita Kaushik, Kavita Gala, Pushpa Singh, S. M. Metkari

**Affiliations:** Stem Cell Biology Department, ICMR - National Institute for Research in Reproductive Health, Jehangir Merwanji Street, Parel, Mumbai, 400 012 India

**Keywords:** Stem cells, VSELs, OCT-4, Endometrium, Estrogen, Progesterone, FSH

## Abstract

**Background:**

Very small embryonic-like stem cells (VSELs) exist in adult organs, express pluripotent markers and have the ability to differentiate into three germ layers in vitro*.* Testicular, ovarian and hematopoietic stem/progenitor cells express receptors for follicle stimulating (FSH) and ovarian hormones and are activated by them to undergo proliferation/differentiation. VSELs exist in mouse uterus and are regulated by physiological dose of estradiol (E) & progesterone (P) during endometrial growth, differentiation and regeneration/remodeling. In the present study, effects of daily administration of E (2 μg/day), P (1 mg/Kg/day) or FSH (5 IU/day) for 7 days on the endometrium and stem/progenitor cells was studied in bilaterally ovariectomized mice.

**Results:**

E treatment resulted in hypertrophy whereas P resulted in hyperplasia and overcrowding of epithelial cells. FSH also directly stimulated the endometrial cells. Nuclear OCT-4A positive VSELs were visualized in ovariectomized (atrophied) endometrium and cytoplasmic OCT-4B positive epithelial, stromal and endothelial cells were observed after treatment. FSH treated uterine tissue showed presence of 4 alternately spliced FSHR isoforms by Western blotting. 3–5 μm VSELs with a surface phenotype of LIN-/CD45-/SCA-1+ were enumerated by flow cytometry and were found to express ER, PR, FSHR1 and FSHR3 by RT-PCR analysis. Differential effects of treatment were observed on pluripotent (Oct4A, Sox2, Nanog), progenitors (Oct-4, Sca-1), primordial germ cells (Stella, Fragilis) and proliferation (Pcna) specific transcripts by qRT-PCR analysis. FSH and P (rather than E) exerted profound, direct stimulatory effects on uterine VSELs. Asymmetric, symmetric divisions and clonal expansion of stem/progenitor cells was confirmed by co-expression of OCT-4 and NUMB.

**Conclusions:**

Results confirm presence of VSELs and their regulation by circulatory hormones in mouse uterus. Stem cell activation was more prominent after P and FSH compared to E treatment. The results question whether epithelial cells proliferation is regulated by paracrine influence of stromal cells or due to direct action of hormones on stem cells. VSELs expressing nuclear OCT-4A are the most primitive and pluripotent stem cells, undergo asymmetric cell division to self-renew and differentiate into epithelial, stromal and endothelial cells with cytoplasmic OCT-4B. Role of follicle stimulating and steroid hormones on the stem cells needs to be studied in various uterine pathologies.

## Background

Uterine endometrium is a highly dynamic tissue that undergoes cyclic cell proliferation, differentiation and remodeling primarily orchestrated by estrogen and progesterone. It is the site of embryo implantation, placentation, fetal development and is affected by diseases like endometriosis and endometrial cancers which are most prevalent gynecological conditions in women. Stem cells are implicated during uterine regeneration/remodeling, maintenance of homeostasis and also pathologies. Presence of stem cells in the uterus was studied earlier by standard approaches like studying label retaining cells and detecting cells in the side population. Research undertaken over last 10 years by the reproductive biologists to identify uterine stem/progenitor cells and their biology was recently reviewed [1–3] and the widely accepted stem cells in the mammalian uterus are possibly the mesenchymal stem cells (MSCs). However, it is still debated whether MSCs are stem cells or just stromal cells [4, 5]. Arnold Caplan, who termed the cells as Mesenchymal Stem Cells in 1996, recently reviewed work done on MSCs and concluded that they are not truly stem cells, rather they arise in vitro from perivascular pericytes and prefers to call them Medical Signaling Cells [6]. High transcriptome similarity exists between cultured pericytes and mesenchymal stromal cells [6]. Taylor et al. [7] suggested a role of bone marrow cells in the regeneration of uterine endometrium. Ong et al. [8] recently showed that bone marrow stem cells do not contribute to endometrial regeneration. Thus the true identity of stem cells in the endometrium remains an open question.

Our group has reported relatively quiescent, pluripotent stem cells termed very small embryonic-like stem cells (VSELs) in the uterine tissue. VSELs exist amongst MSCs from various sources [9] and it is very likely that these stem cells, being of very small size and scarce in nature, have been missed by various investigators while studying endometrial MSCs. VSELs were reported in adult mouse uterus; they survive in atrophied bilaterally ovariectomized mouse uterus and are regulated by physiological doses of sex hormones to mediate proliferation, receptive stage as well as remodeling/ regeneration [10]. These stem cells have also been reported in myometrium as well as in perimetrium and could be a likely source of tumor initiating cells leading to formation of leiomyomas [11]. Xiao et al. [12] have reported up- regulation of Oct-4, Sox2 and Nanog (specific markers for pluripotent VSELs) in LPS model of acute uterine injury and also report presence of these markers in human endometrium and up-regulation in women with intrauterine adhesions at both mRNA and protein level. Although not reported specifically as VSELs, indirect evidence suggests that pluripotent cells (VSELs) do exist in human endometrium and may be implicated in various pathologies including endometriosis (Table 1) [13–22]. As evident it is crucial to selectively analyze OCT-4A to arrive at meaningful data since alternatively spliced isoforms OCT-4A and OCT-4B have indeed confused the field as suggested earlier [23, 24]. VSELs in the bone marrow get mobilized under stress conditions and have confused investigators who reported bone marrow as a source of germ cells in females [25], males [26] and also in the endometrium [7].

**Table 1:** Details of primers used for various experiments

Gene Names	Primer Sequence	Annealing Temp (°C)	Amplicon Size (bp)
Pcna	F:GATGCCGTCGGGTGAATTTG R:TCTCTATGGTTACCGCCTCCT	55	182
Oct-4	F:CCTGGGCGTTCTCTTTGGAAAGGTG R: GCCTGCACCAGGGTCTCCGA	62	177
Oct-4A	F: CCATGTCCGCCCGCATACGA R: GGGCTTTCATGTCCTGGGACTCCT	60	190
Nanog	F: CAGGAGTTTGAGGGTAGCTC R: CGGTTCATCATGGTACAGTC	61	223
Sox-2	F:GGGGGAAAGTAGTTTGCTGCCTCT R:TGCCGCCGCCGATGATTGTT	61	135
FSHR1	F:CATTCACTGCCCACAACTTTCATC R:TGAGTGTGTAATTGGAACCATTGGT	65	256
FSHR3	F:TCTCCACTGCTGCACTGTTGGGCT R:ATTCAAATACAGGAAATAGAGAAA	56	111
Sca-1	F:AGAGGAAGTTTTATCTGTGCAGCCC R:TCCACAATAACTGCTGCCTCCTGA	58	223
Stella	F:ACGCTTTGGATGATACAGACGTCC R:GCGCTTTGAACTTCCCTCCGGA	59	175
Fragallis	F:GGGGTGACTGAGCTGGGGGAA R:TGTCCCTAGACTTCACAGAGTAGGC	61	178
ER	F:CCTTCTAGACCCTTCAGTCAAGC R:CGAGACCAATCATCAGAATCTCC	65	155
PR	F: CCAGCTCACAGCGCTTCTACC R: GAAAGAGGAGCGGCTTCACC	62	198
18S	F:GGAGAGGGAGCCTGAGAAAC R: CCTCCAATGGATCCTCGTTA	60	171

VSELs exist in various adult organs and serve as backup pool for adult tissue specific progenitors or 'stem cells' throughout life and were recently reviewed [27, 28]. VSELs express pluripotent markers and have the ability to differentiate into 3 germ layers in vitro both in humans [29–31] and mice [32, 33]. Shaikh et al. [32] also reported differentiation of bone marrow VSELs into male germ cells when cultured on a Sertoli cells bed. Recent published data suggests that VSELs along with spermatogonial stem cells in testis, ovarian stem cells in ovary and hematopoietic stem cells in cord blood and bone marrow express receptors for gonadotropin and steroid hormones. Patel et al. [34] showed expression of FSHR on ovarian stem cells and their activation by FSH. Mierzejewska et al. [35] and Abdelbaset-Ismail et al. [36] have reported expression of sex hormone receptors on hematopoietic stem cells. Patel and Bhartiya [37] reported expression of FSHR on testicular stem cells. This similar expression of FSHR on stem cells in the hematopoietic system as well as in the gonads suggests their common developmental link with primordial germ cells (PGCs) and was recently discussed [28]. It has been postulated that PGCs exist in various adult organs in few numbers as VSELs.

Present study was undertaken to study the treatment effects of estradiol, progesterone and FSH on uterine stem/progenitor cells. Response to treatment was evaluated on H&E stained sections and by studying the expression of PCNA (suggestive of proliferation) and OCT-4 (stem cell marker). ER, PR and FSHR expression was studied on an enriched population of stem cells. FSHR expression on uterine tissue was further confirmed by Western blotting. Treatment effects were also assessed by flow cytometry to enumerate 3–5 μm VSELs with a surface phenotype of LIN-/CD45-/SCA-1+ and qRT-PCR analysis of transcripts specific for pluripotent state (Oct4A, Sox2, Nanog); progenitors (Oct-4, Sca-1); primordial germ cells (Stella, Fragilis) and proliferation (Pcna). Higher dose of FSH/steroids used in the present study allowed deciphering stem cells activity in the endometrium. Mice were not sensitized with low dose of estrogen prior to treatment and the treatment used in the present study helps achieve levels normally observed during pregnancy [11].

## Methods

The study was approved by institute stem cells and animal ethics committees. Bilateral ovariectomy was performed on eight weeks old Swiss mice and after14 days; they were treated with hormones [estrogen 2 μg/day; progesterone 1 mg/Kg for 7 days; FSH 5 IU/day for 5 days]. Uterine tissue (6 mice per group) was collected and appropriately processed for histological studies, immuo-histochemistry, Western blotting, RNA extraction and for flow cytometry studies. Tissue was fixed in neutral buffered formalin and standard protocols were used to prepare paraffin blocks; serial sections were cut and stained with Hematoxylin & Eosin (H&E). Sections were viewed and representative areas were recorded using NIKON 90i Bright field microscope.

### Immuno- localization of OCT-4 and PCNA and NUMB

OCT-4 antibody (ab19857, ABCAM, UK, raised from within residues 300 to the C-terminus of human Oct-4) allowed identification of both the alternatively spliced isoforms of OCT-4. Nuclear OCT-4A is crucial to maintain pluripotent state and as the cell initiates differentiation, OCT-4B is expressed in the cytoplasm and eventually gets degraded and is lost in mature cells [28]. Similar nuclear and cytoplasmic OCT-4 localization in pluripotent and non-pluripotent human primordial germ cells (PGCs) has been reported by others also [38]. Proliferating cell nuclear antigen (PCNA) is a surrogate marker to study mitogenic effect and monoclonal anti-PCNA mouse IgG antibody (P8825, Sigma) was used in the present study to gauge the effect of treatment on proliferation of endometrial cells. OCT-4 (ab4419, Millipore, USA; specific to OCT-4A) was used along with NUMB (ab14140, ABCAM; specific to progenitors that arise by asymmetric cell divisions, ACD from stem cells) antibody to study stem cell divisions.

Briefly, the paraffin embedded uterine tissue sections were deparaffinized and incubated in xylene for 30 mins after air drying slides were incubated with 3% hydrogen peroxide (Qualigens, India) in 100% methanol for 30 mins in dark after which the sections were gradually hydrated in descending series of methanol to tap water for 5 min each. This was followed by antigen retrieval by immersing the slides in boiling sodium citrate (SSC, Sigma) buffer at pH 6 for 5 mins. After cooling, the slides were washed with water and 1X PBS buffer for 5 mins each. Permeabilization of the sections was done with 0.3% TritonX-100 in PBS buffer for 10 mins for OCT-4. Blocking was done with 10% NGS for 2 h for OCT-4 antibody raised in rabbit and along with 10% normal horse serum for PCNA antibody raised in mice, followed by incubation with the primary antibody OCT-4 (1:100) and PCNA (1:3000) at 4 °C overnight. Primary antibody was replaced with blocking solution for negative control. Next day slides were washed 3 times with PBS (5 min each wash) and then incubated with respective biotinylated secondary antibody for 30 mins followed by avidin biotin complex formation step for 30 min (Vectastain Elite ABC kit, Vector Laboratories Inc., USA), 3 washes with PBS and then color reaction was done using diaminobenzidene (Biogenex, USA). The slides were then counterstained with Hematoxylin, dehydrated and cover slipped. Representative areas were photographed under Nikon 90i microscope and the data was recorded. Co-expression of NUMB and OCT-4 was studied on endometrial cells using protocols and imaged using confocal microscope as reported earlier for bone marrow stem cells [39].

### Western blotting

FSH treated mouse uterine tissue was lysed in RIPA buffer (Thermo Scientific, USA) along with 0.2 mM PMSF (Sigma Aldrich) and 25X protease inhibitor (Roche Diagnostics, Germany). The cell lysate was sonicated in an Ultrasonicator (Oscar Ultrasonics Pvt. Ltd., India) for 5 min followed by incubation on ice for 30 min and then centrifuged to collect the supernatant. 300 μg of protein was loaded onto 12% SDS-PAGE followed by transfer onto Nitrocellulose membrane (Amersham Biosciences, UK). The blot was blocked with 5% dried milk in 1X Tris buffered saline (TBS, 10 mM Tris,150 mM NaCl, pH 7.6) for 1 h and then incubated overnight at 4C in primary rabbit anti FSHR (1:250Abcam) antibody raised against the N-terminal extracellular domain of FSHR which is conserved in all the reported FSHR isoforms [40]. After washes to remove unbound antibody, the membrane was incubated with goat anti-rabbit IgG HRP conjugated secondary antibody (1:1000, Invitrogen, USA) for 2 h at RT. The blot was imaged using Super Signal West Femto substrate (Thermo Scientific, USA).

### Sample preparation for making cell smears and for RNA studies

Stem cells were enriched from progesterone treated uterus. For this, the uterine tissue was enzymatically digested using collagenase IV (1 mg/ml), trypsin (1 mg/ml) and DNAse (0.5 mg/ml) at 37 C for 45 min. After adding 20% fetal bovine serum, cells suspension was filtered through a 40 μm filter to remove all the undigested debris and then spun at 200 g for 10 min. Majority of somatic cells settled down as a big pellet. Supernatant was further spun at a higher speed of 1000G. This led to the pelleting and relative enrichment of stem/progenitor cells [28, 41]. These stem/progenitor cells were used to make cell smears and were also placed in TRIzol for RNA extraction to study expression of ER, PR and FSHR (FSHR1 and FSHR3). In addition, uterine tissues from various treatment groups (ovariectomized, E, P and FSH) were also placed in TRIzol for RNA extraction to study differential expression of pluripotent (Oct-4A, Sox2, Nanog), proliferation (Pcna), progenitor (Sca-1, Oct-4) and FSH receptor isoforms (R1 and R3) transcripts by qRT-PCR. List of primers used for various experiments are provided in Table 1.

### RNA extraction, cDNA synthesis, RT-PCR and qRT-PCR

#### RNA extraction and cDNA synthesis

Total RNA was isolated from cell pellets/tissue samples in TRIzol, according to manufacturer’s instructions. First-strand cDNA was synthesized using the iScript cDNA synthesis Kit (Bio-Rad, USA) according to the manufacturer’s instructions in G-STORM thermocycler (Gene Technologies, UK).

#### Reverse transcriptase -PCR studies

RT-PCR was carried out to study the expression of ER, PR and FSHR on an enriched population of stem/progenitor cells. Briefly, the cDNA mix (2 μl) was amplified using 0.2 mM of each primer, 1.25 unit of DreamTaq DNA polymerase (Fermentas Life Sciences; Lithuania) in 1× buffer and 0.2 mM dNTPs in a G-STORM thermocycler. Amplification was carried out for 35 cycles, with each cycle consisting of denaturation at 94 °C for 30 s, annealing at the specified temperature for 20 s, and extension at 72 °C for 30 s. The products were analyzed on 2% agarose gel stained with 0.5 μg/ml ethidium bromide. The product size was approximated using a 100-bp DNA ladder (Bangalore Genei, India). The negative control did not include cDNA in the reaction mixture.

#### Quantitative RT-PCR studies

The expression levels of various transcripts were estimated by CFX96 real-time PCR system (Bio-Rad Laboratories, USA) using SYBR Green chemistry (Bio-Rad). 18 s was used as housekeeping gene. The amplification conditions were initial denaturation at 94 °C for 3 min followed by 40 cycles comprising of denaturation at 94 °C for 10 s, annealing for 20 s, and extension at 72 °C for 30 s followed by melt curve analysis. The fluorescence emitted was collected during the extension step of each cycle. The homogeneity of the PCR amplicons was verified by running the products on 2% agarose gels and also by studying the melt curve. All PCR amplifications were carried out in duplicate. Mean Ct values generated in each experiment using the CFX Manager software (Bio-Rad) were used to calculate the mRNA expression levels. The fold change was calculated using ΔΔCt method. The relative expression levels of each gene were compared between various treatment groups and ovariectomized mouse uterus that was used as reference sample.

### Flow cytometry analysis of stem cells

Flow cytometry was used for enumerating VSELs (2–5 um cells with a surface phenotype of LIN-/SCA-1+/CD45-) in uterine tissue after various treatments. A single cell suspension was prepared and stained as described earlier [11]. Briefly, the final cell suspension in 1 ml is stained with fluorescein isothiocyanate conjugated rat anti-mouse SCA-1 (1 μg/million cells, BD Biosciences, USA), phycoerythrin conjugated rat anti-mouse CD45 (2 μg/million cells, BD) and allophycocyanin conjugated mouse lineage antibody cocktail (25 μl/ml cells, BD). After washing with PBS and centrifugation at 1000 g for 10 min, the stained cells were run on FACS Aria (BD Biosciences, San Jose, CA, USA). Results were analyzed using FACS Diva software (BD Biosciences).

### Stem cells proliferation kinetics

To clearly demonstrate the heterogeneity amongst uterine stem cells, various cells were arranged on the basis of their size and pattern of cell divisions as reported recently for various tissues [42].

## Results

### Histological changes in atrophied endometrium and in response to E, P & FSH treatment

#### Atrophied mouse endometrium (2 weeks after bilateral ovariectomy)

**Fig. 1:**
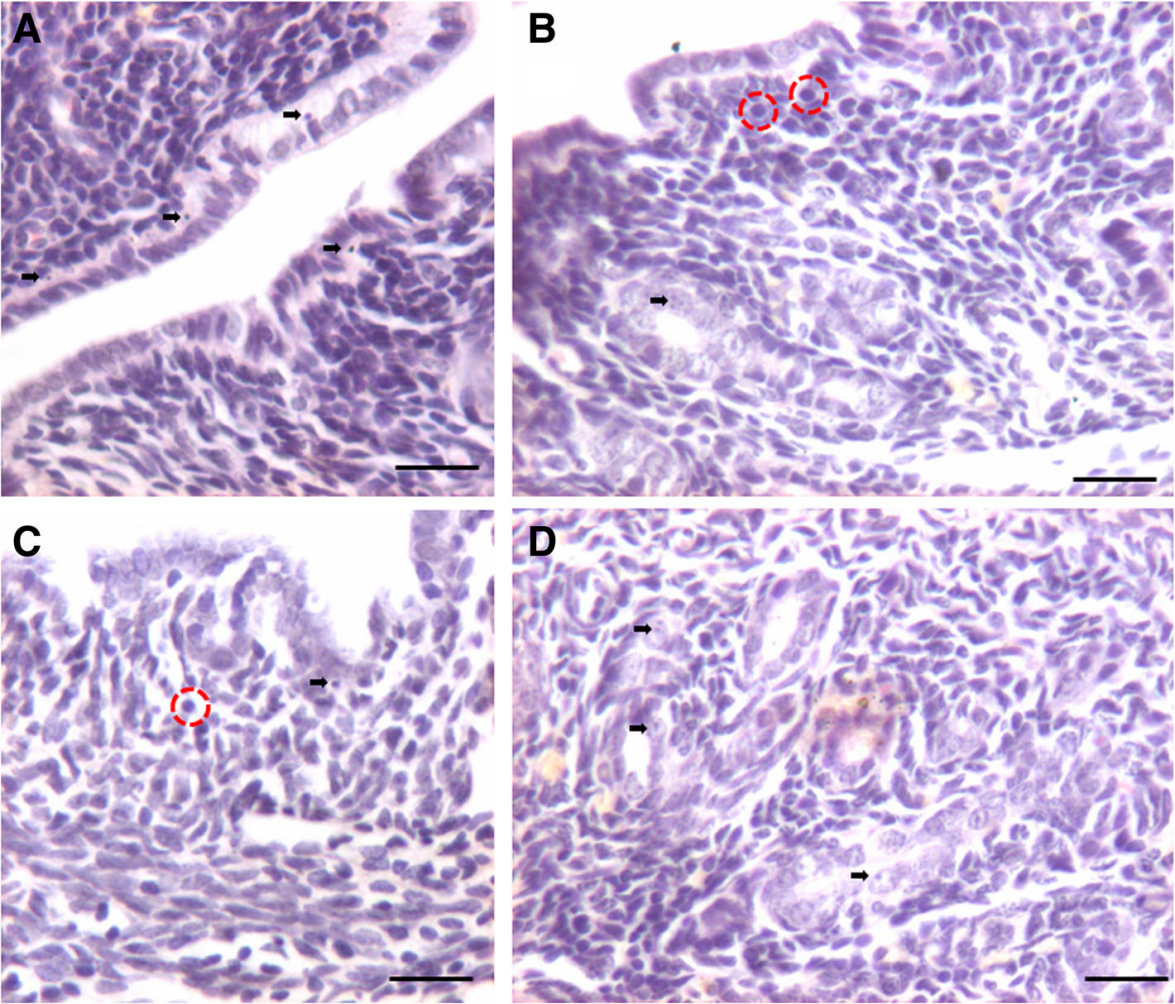
Bilaterally ovariectomized mouse uterine sections after H&E staining. **a**-**d** The cells lining the luminal epithelium and the glands as well as the stromal cells are atrophied in nature with minimal pink stained cytoplasm and high nucleo-cytoplasmic ratio. A careful examination shows the presence of small spherical cells (arrow) and also slightly bigger spherical cells (broken circle). In the luminal epithelium, these small spherical cells are located along the basal region of epithelial cells. Scale bar 20 μm

Histological analysis of mouse uterus showed completely atrophied endometrium (Fig. 1). Luminal epithelial cells were small in size, cuboidal in shape with high nucleo-cytoplasmic ratio and minimal pink cytoplasm. Glands were small in size and stromal compartment was compact comprising cells with minimal cytoplasm. A careful examination of luminal and glandular epithelium showed the presence of distinct spherical cells (of two distinct sizes) with darkly stained nucleus. Small cells appeared to be of different size possibly because they were cut in different planes. These spherical, putative stem cells were located in the basal region of luminal epithelium and at places on top of the nuclei of epithelial cells. It has been reported earlier that these stem cells survive in close proximity of or within other cells by a process termed ‘emperopolesis’ [43]. We could detect the putative stem cells in atrophied endometrium because we knew what to look for after having published extensively on these stem cells in other organs [28] as well as in the uterus [10, 11]. Main characteristic features of these stem cells included their small size, spherical shape, dark stained nucleus, minimal cytoplasm and high nucleo-cytoplasmic ratio.

#### Effect of estrogen treatment (2 μg/day for 7 days)

**Fig. 2:**
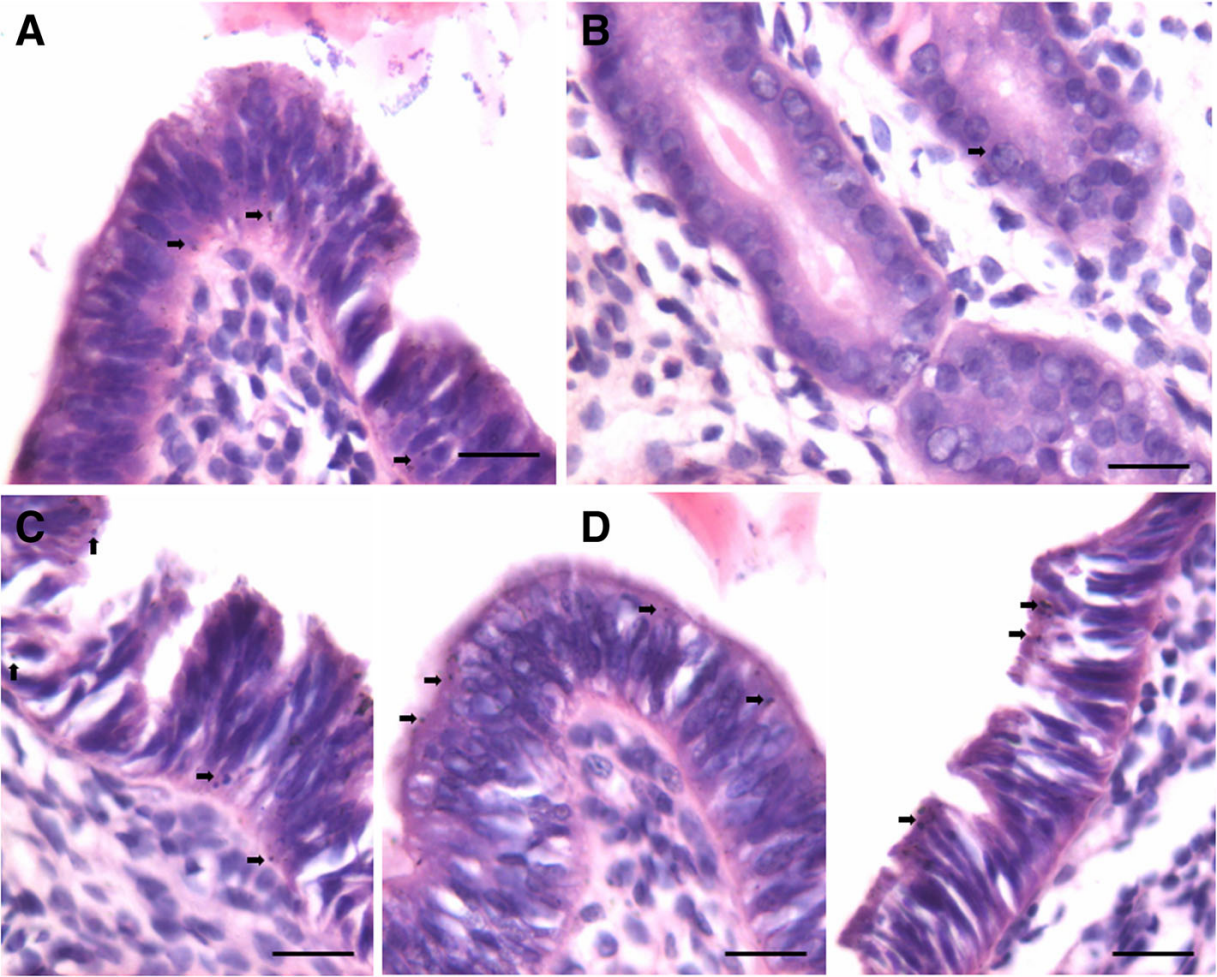
Effect of estrogen (2 μg/day for 7 days) treatment to bilaterally ovariectomized mice. **a**, **b**, **c**, **d**. H&E stained uterine sections show marked hyperptrophy of epithelial cells lining the lumen as well as the glands was noted. Cells increased in height and exhibit abundant pink cytoplasm. Small spherical cells were visualized (arrow) similar to in the ovariectomized group. Scale bar 20 μm

Estrogen treatment resulted in marked hypertrophy of cells lining both luminal and glandular epithelium with abundant pink cytoplasm in H&E stained sections (Fig. 2). Luminal epithelial cells appeared to be multilayered and putative stem cells were visualized along the basal region of epithelial cells. Glands were bigger in size and with abundant pink cytoplasm. Stromal cells appeared more developed and pink compared to ovariectomized group (Fig. 1) with altered shape and lot of edema.

#### Effect of progesterone treatment (1 mg/kg per day for 7 days)

**Fig. 3:**
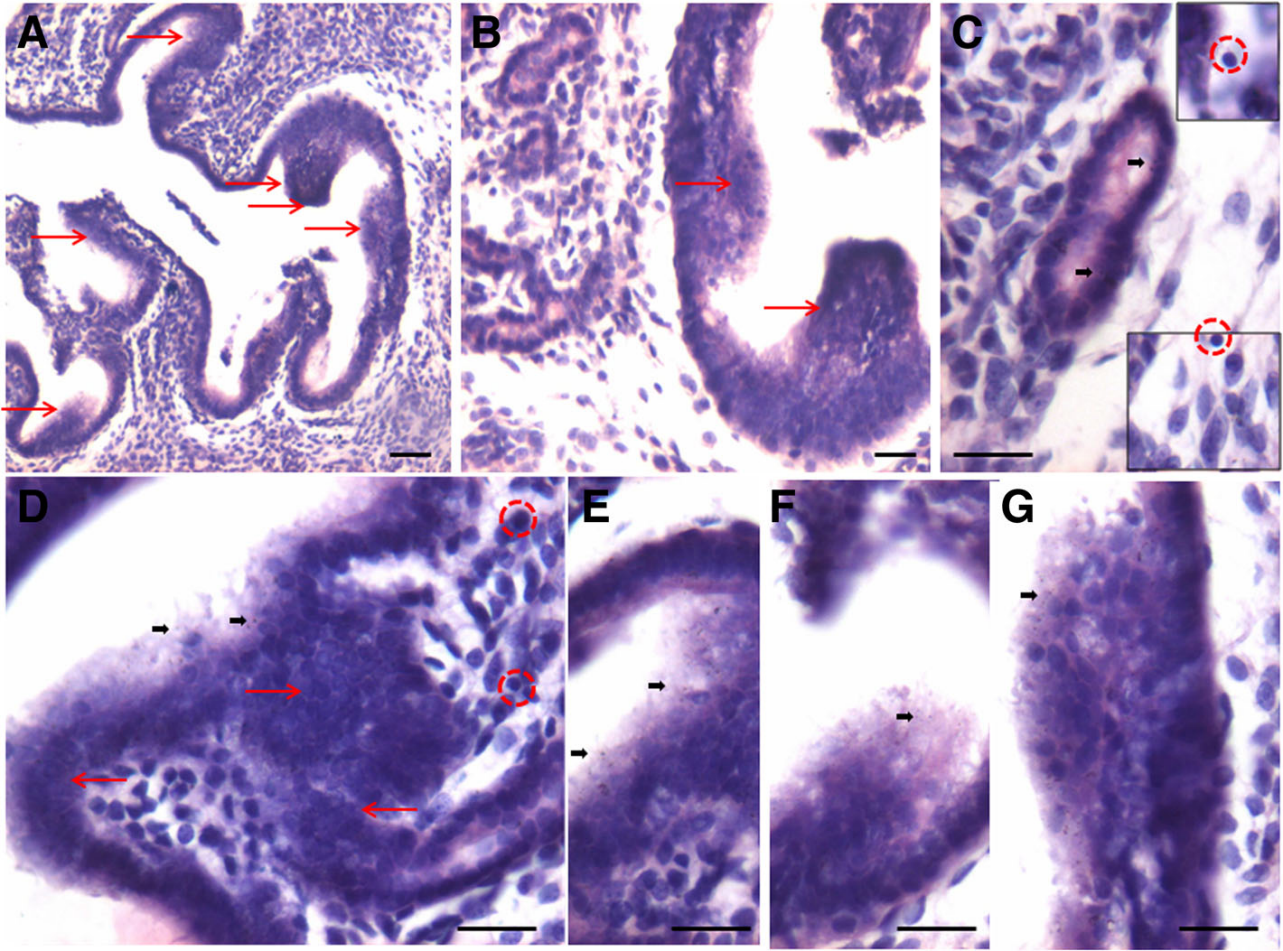
Effect of progesterone (1 mg/Kg/day for 7 days) treatment to bilaterally ovariectomized mice. **a**, **b**, **c**, **d**, **e**, **f**, **g**. H&E stained uterine sections show a marked overcrowding of epithelial cells was noted lining the lumen suggesting hyperplasia of cells (red arrows) with high nucleo-cytoplasmic ratio and minimal cytoplasm. Small (black arrow) and slightly bigger (broken circle) spherical putative stem cells were clearly visualized. Note that despite excessive turnover of cells, mitotic figures are not visualized. Scale bar 20 μm

**Fig. 4:**
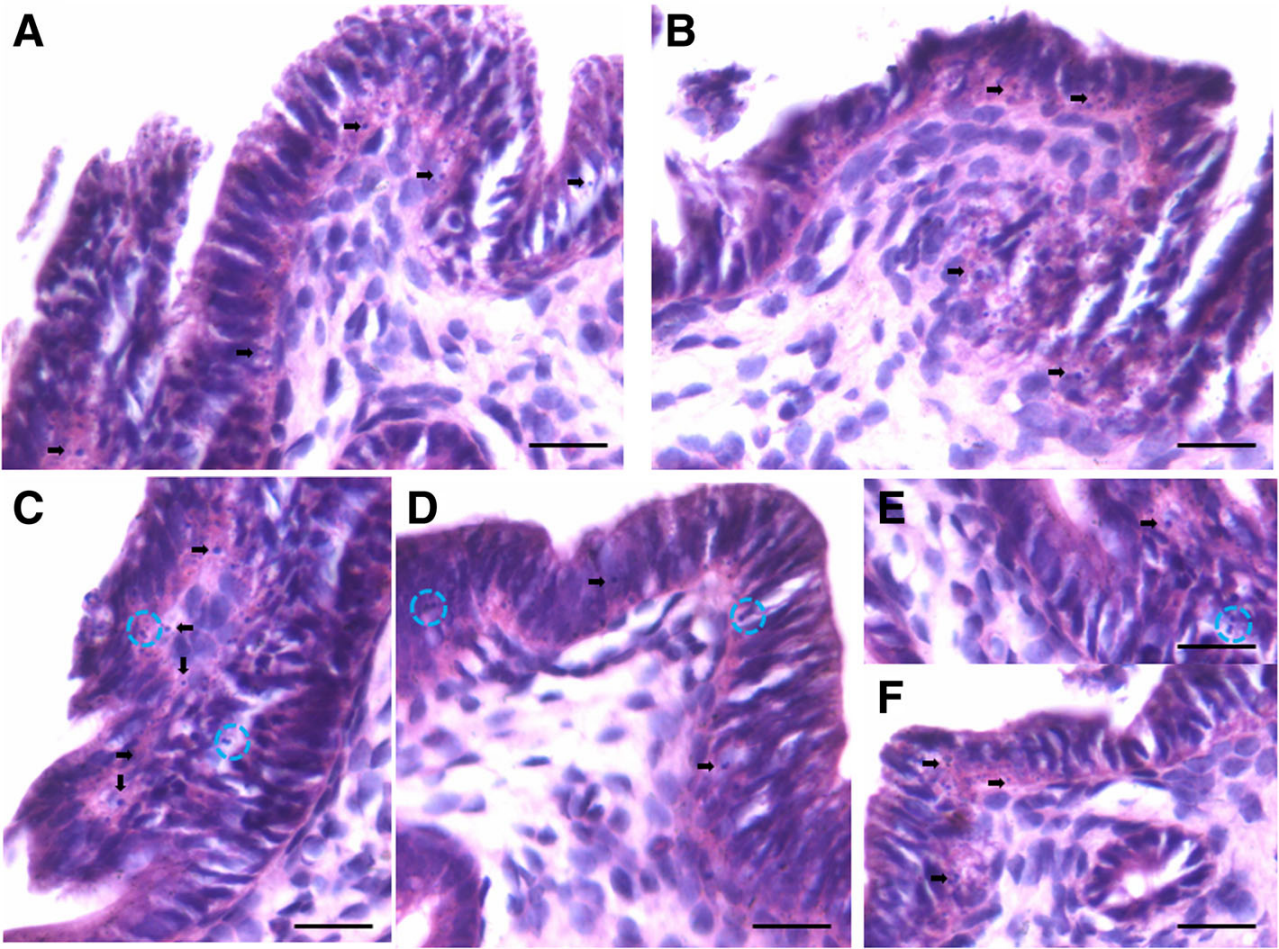
Effect of follicle stimulating hormone (5 IU/day for 5 days) treatment to bilaterally ovariectomized mice. **a**, **b**, **c**, **d**, **e**, **f**. H&E stained uterine sections show hypertrophy of epithelial cells and the spherical cells (arrow) were observed in large numbers. At few places, it appeared as though these spherical cells were dividing (broken blue circle). Compared to E (Fig. 2) & P (Fig. 3) treatment, numbers of spherical cells were more clearly evident after FSH treatment. Scale bar 20 μm

Endometrial response to P treatment was remarkable (Fig. 3) in the absence of prior estrogen priming to induce PR expression. Majority of cells appeared blue in H&E stained sections with very little pink stained cytoplasm. Epithelial cells remained small in size but revealed extensive hyperplasia. Remarkable proliferation and overcrowding of cells was clearly evident. Stromal cells also remained compact. Small stem cells were clearly evident (black arrow) and slightly bigger progenitor cells were also visualized (encircled). Stromal cells were overcrowded and glands appeared different compared to those observed after estrogen treatment. Fig. 3d shows extensive overcrowding of luminal epithelium and how these cells move into the stromal to give rise to glandular epithelium. Surprisingly we did not see any mitotic figures amongst the epithelial cells, despite their excessive turnover and overcrowding (Fig. 4).

#### Effect of treatment with follicle stimulating hormone (5 IU/day for 5 days)

Effect of FSH treatment on mouse endometrium (Fig. 4) was interesting and unexpected, however we were not surprised as FSHR expression has been ubiquitously expressed on various organs [44] including various reproductive tissues [45]. A distinct hypertrophy of epithelial cells was observed but was relatively less compared to that observed after estrogen treatment (Fig. 2). Glands remained small and stromal cells also responded to FSH treatment and revealed lot of pink cytoplasm compared to stromal cells in atrophied endometrium (Fig. 1). Spherical, putative stem cells were clearly visualized in large numbers located majorly in the basal region of luminal epithelium. At places, dividing stem cells were also observed (encircled).

To conclude, a significant atrophy of endometrium occurs when deprived of steroid hormones (bilateral ovariectomy), treatment with estrogen results in hypertrophy of epithelial cells, progesterone treatment resulted in overcrowding and hyperplasia of epithelial cells (suggestive of extensive stem cells activity) and FSH also exerted direct effect on endometrial histology. Maximum numbers of stem cells were visualized in H&E stained sections after FSH treatment. In order to further confirm the effect of treatment on proliferation/ differentiation, immuno-localization of PCNA, a surrogate marker for proliferation was undertaken.

### PCNA expression in atrophied endometrium and in response to E, P and FSH treatment

**Fig. 5:**
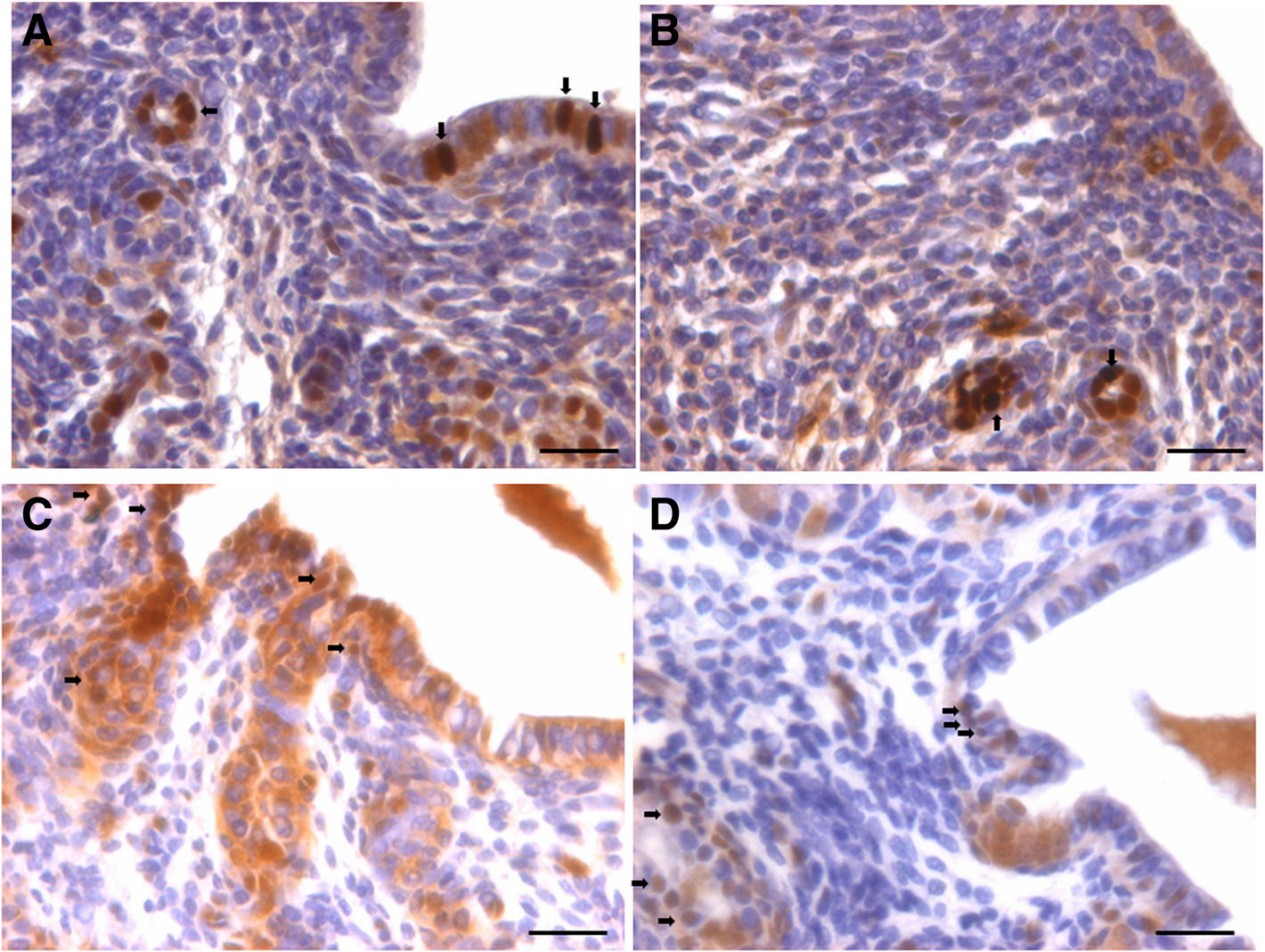
PCNA and OCT-4 expression in bilaterally ovariectomized mouse endometrial section. **a**, **b**. PCNA expression was minimal in the epithelial cells lining the lumen and the glands. Stromal cells did not express PCNA. Please note that few cells show relatively intense expression compared to others (arrow). **c**, **d**. OCT-4 expression was cytoplasmic in nature however; few spherical cells (arrow) expressed nuclear OCT-4A. Thus the OCT-4 antibody used in the present study helps to clearly delineate the two alternatively spliced isoforms of OCT-4 including nuclear OCT-4A and cytoplasmic OCT-4B. Scale bar 20 μm

**Fig. 6:**
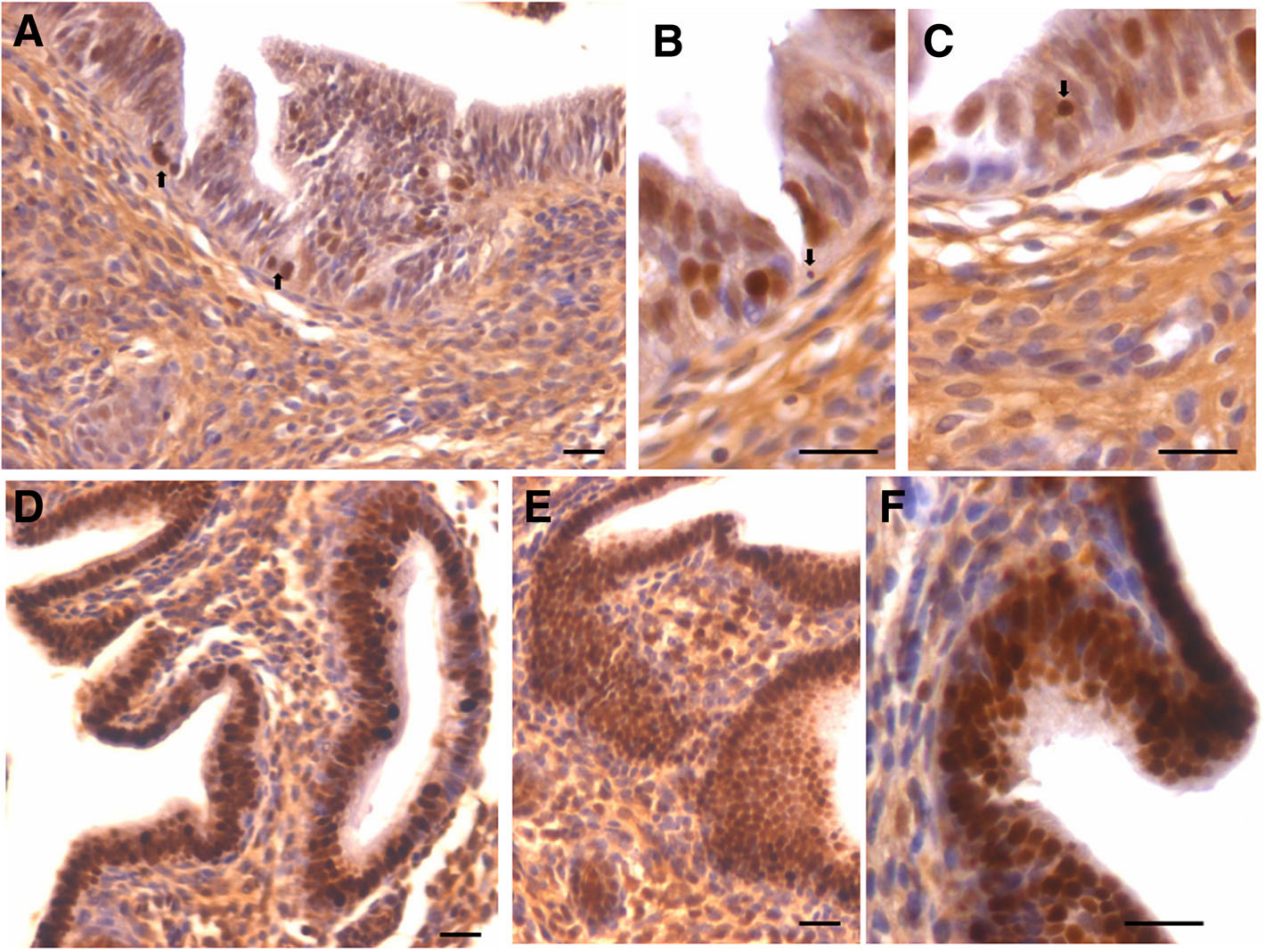
PCNA expression in endometrial sections after estrogen and progesterone treatment to bilaterally ovariectomized mice. **a**, **b**, **c** Large number of cells showed nuclear PCNA expression suggestive of increased proliferation after E treatment however. At the same time, large number of cells had blue nuclei stained negative for PCNA. Small spherical cells with PCNA positive nuclei were clearly visualized (arrow). **d**, **e**, **f** Markedly increased PCNA expression was observed in both luminal and glandular epithelial cells after P treatment. All the nuclei in the luminal epithelium were stained positive for PCNA. However, none of the cells show any mitotic figure which is indeed surprising. This observation leads us to believe that epithelial cells arise by differentiation of stem cells and that proliferation/clonal expansion is restricted to the stem cells compartment (please refer to Fig. 12). Non-specific, excessive brown color in the sections is because of endogenous mouse Ig staining since we used mouse primary antibody on mouse tissue. Scale bar 20 μm

**Fig. 7:**
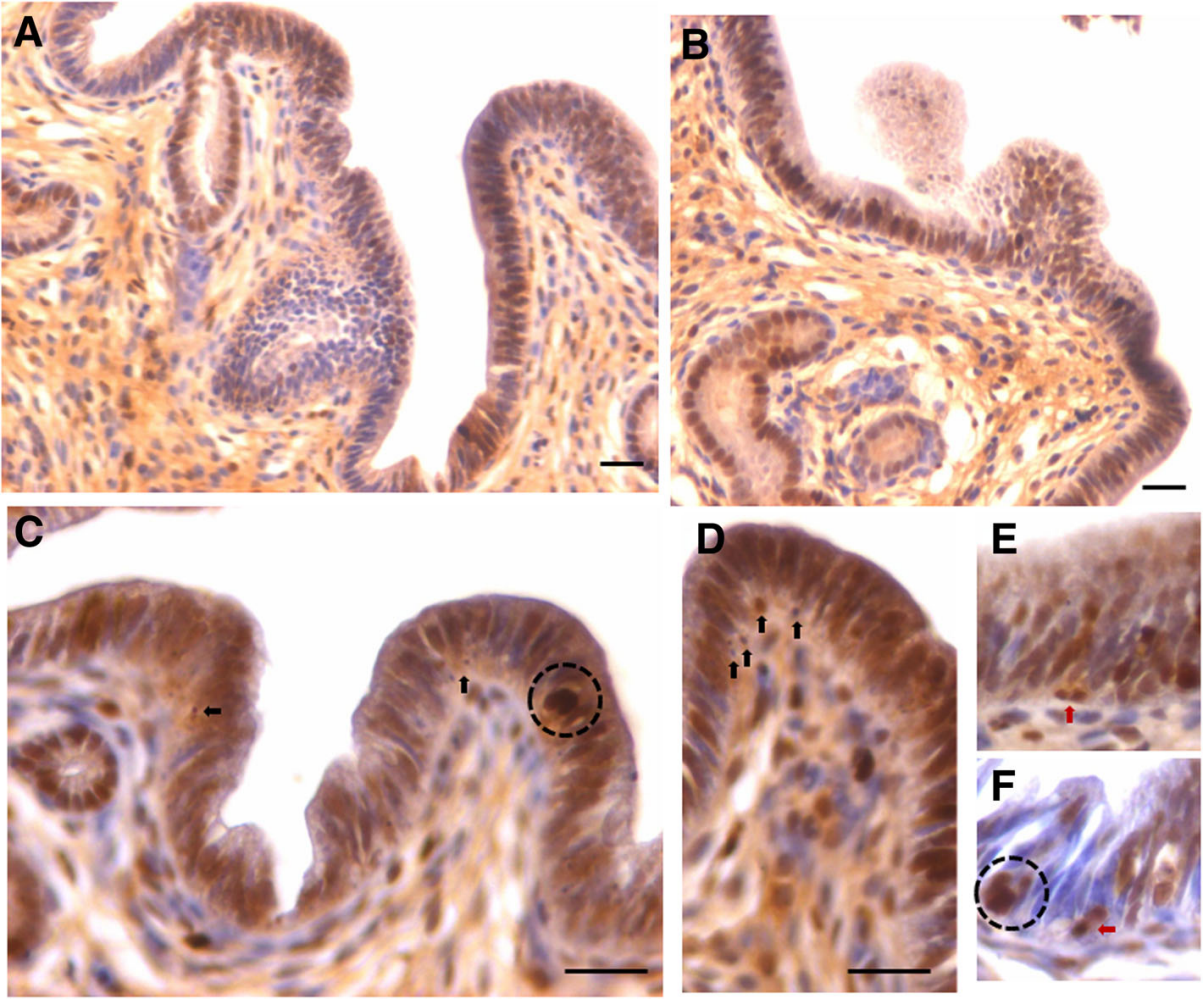
PCNA expression in endometrial sections after follicle stimulating hormone treatment to bilaterally ovariectomized mice. **a**, **b**. PCNA expression after FSH treatment was similar to after E treatment in the epithelial cells implying few cells were positive and few were negative for PCNA. **c**, **d**, **e**, **f**.  At higher magnification one could observe the small spherical cells expressing PCNA (black arrow) and these cells were also evidently dividing (red arrow). It is interesting to note that mitotic figures are not observed in the epithelial cells. Note two clump of cells (broken circle) in the basal region possibly suggestive of clonal expansion of stem cells. This becomes very clear on studying stem cell smears (Fig. 12). Scale bar 20 μm

Minimal PCNA expression was observed in few cells lining luminal as well as glandular epithelium in ovariectomized uterus whereas majority of epithelial and stromal cells remained negative (Fig. 5, upper panel). Distinct cells expressing nuclear PCNA were observed in the luminal and glandular epithelium and we could hardly observe stromal cells expressing PCNA after estrogen treatment (Fig. 6, upper panel). PCNA expressing cells were in much larger numbers after treatment with P (Fig. 6, lower panel). FSH treatment also resulted in PCNA expression in epithelial cells (Fig. 7).

It was interesting to note variable intensity of PCNA expression in epithelial cells in various treatment groups. In ovariectomized group, few epithelial cells had intense PCNA expression compared to others. In E treated group, besides oval shaped nuclei in epithelial cells showing faint PCNA expression, there were distinct spherical cells with intense brown PCNA expression. Interestingly these dark stained spherical cells were present along the basal region of the epithelial cells and were distinctly visualized after various treatments (Figs. 6 and 7, lower panel). Despite excessive turnover of cells, we did not observe mitotic figures and this observation leads us to propose that epithelial cells possibly arise by differentiation of stem cells and that proliferation/clonal expansion is restricted to the stem cells compartment (Fig. 12).

To conclude, PCNA expression increased after treatment with maximum hyperplasia observed after progesterone treatment. Also FSH exerted a marked effect on PCNA expression in the epithelial cells. Distinct populations of small sized, spherical ‘putative stem’ cells with intense PCNA expression were observed along the basal layer. They were of variable size possibly because of different plane of section through the stem cells. PCNA expression was observed in the epithelial cell nuclei however no mitotic figures were observed among the epithelial cells.

### OCT-4 expression in atrophied endometrium and in response to E, P and FSH treatment

**Fig. 8:**
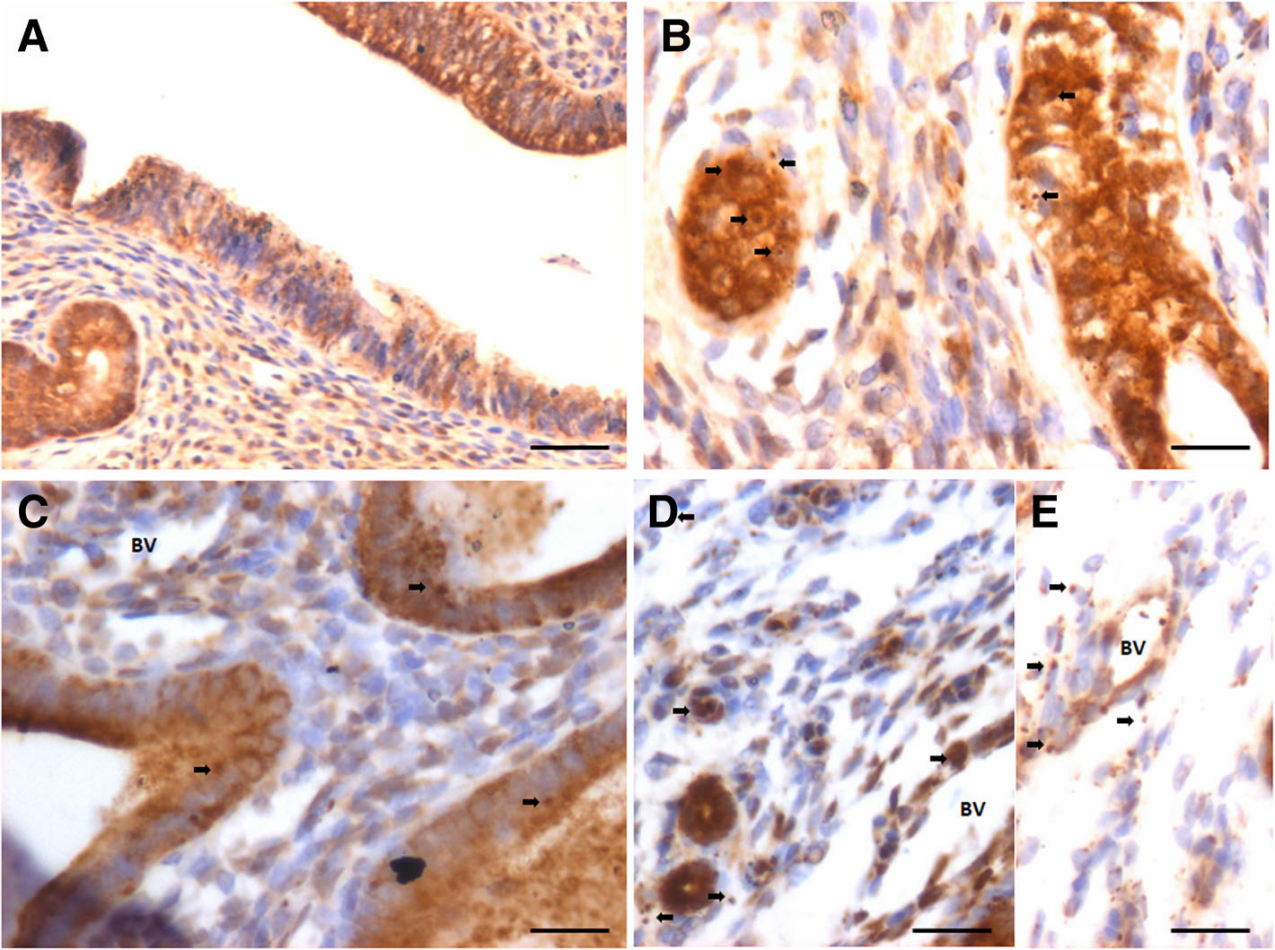
OCT-4 expression after E (**a**, **b**) and P (**c**, **d**, **e**) treatment to bilaterally ovariectomized mice. OCT-4 expression was primarily in the cytoplasm of epithelial cells after E and P treatment. Few spherical cells expressing nuclear OCT-4A were also visualized (arrow). Endothelial cells lining the blood vessels (BV) also expressed cytoplasmic OCT-4. Scale bar 20 μm

**Fig. 9:**
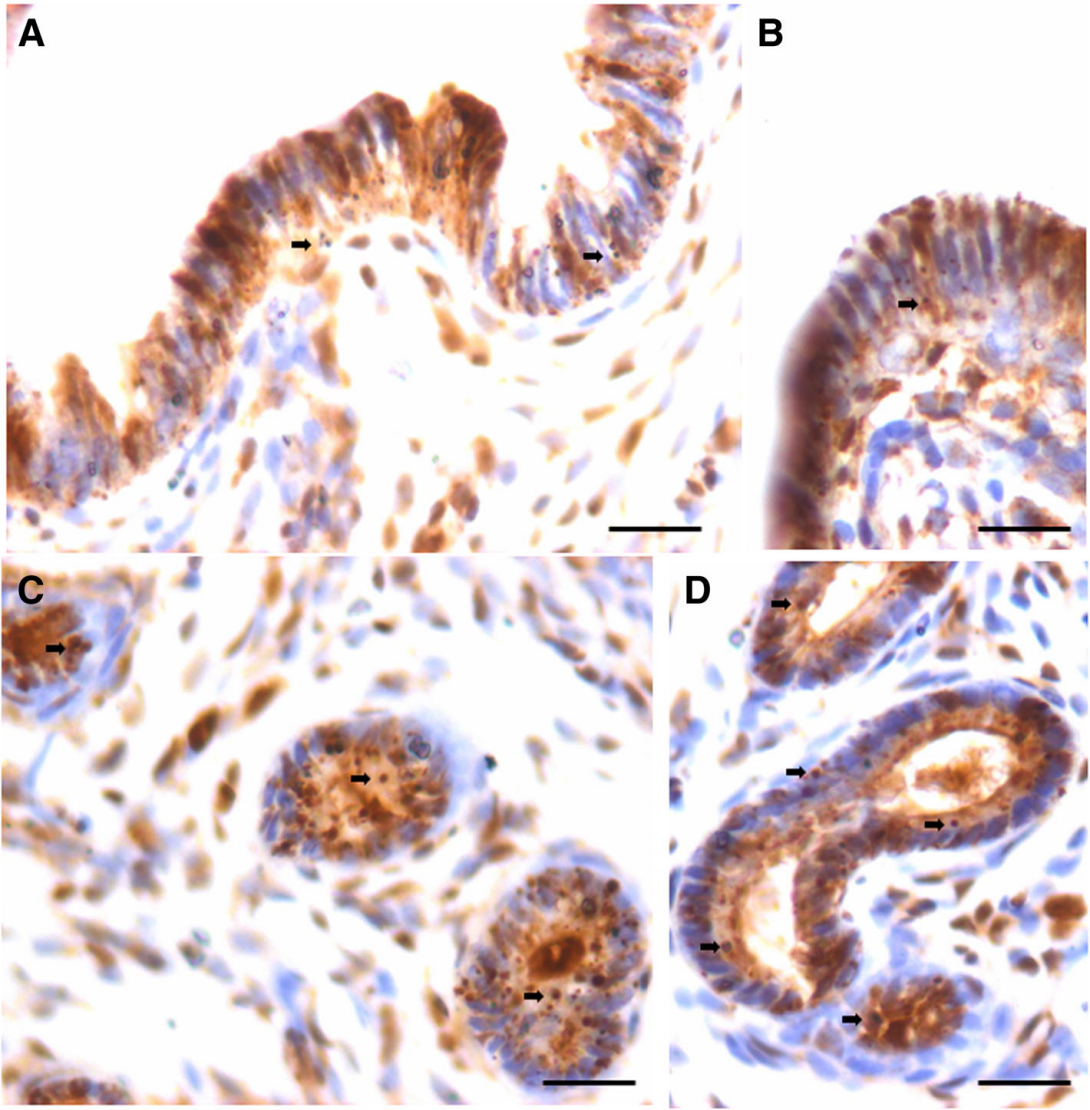
OCT-4 expression after treatment with follicle stimulating hormone to bilaterally ovariectomized mice. **a**, **b**, **c**, **d**. OCT-4 expression was noted prominently in the cytoplasm of epithelial cells whereas small spherical cells expressed nuclear OCT-4. Stromal cells also expressed cytoplasmic OCT-4. Large numbers of spherical stem cells expressing OCT-4 were observed in the glands however the glandular epithelial cells remained negative. Scale bar 20 μm

**Fig. 10:**
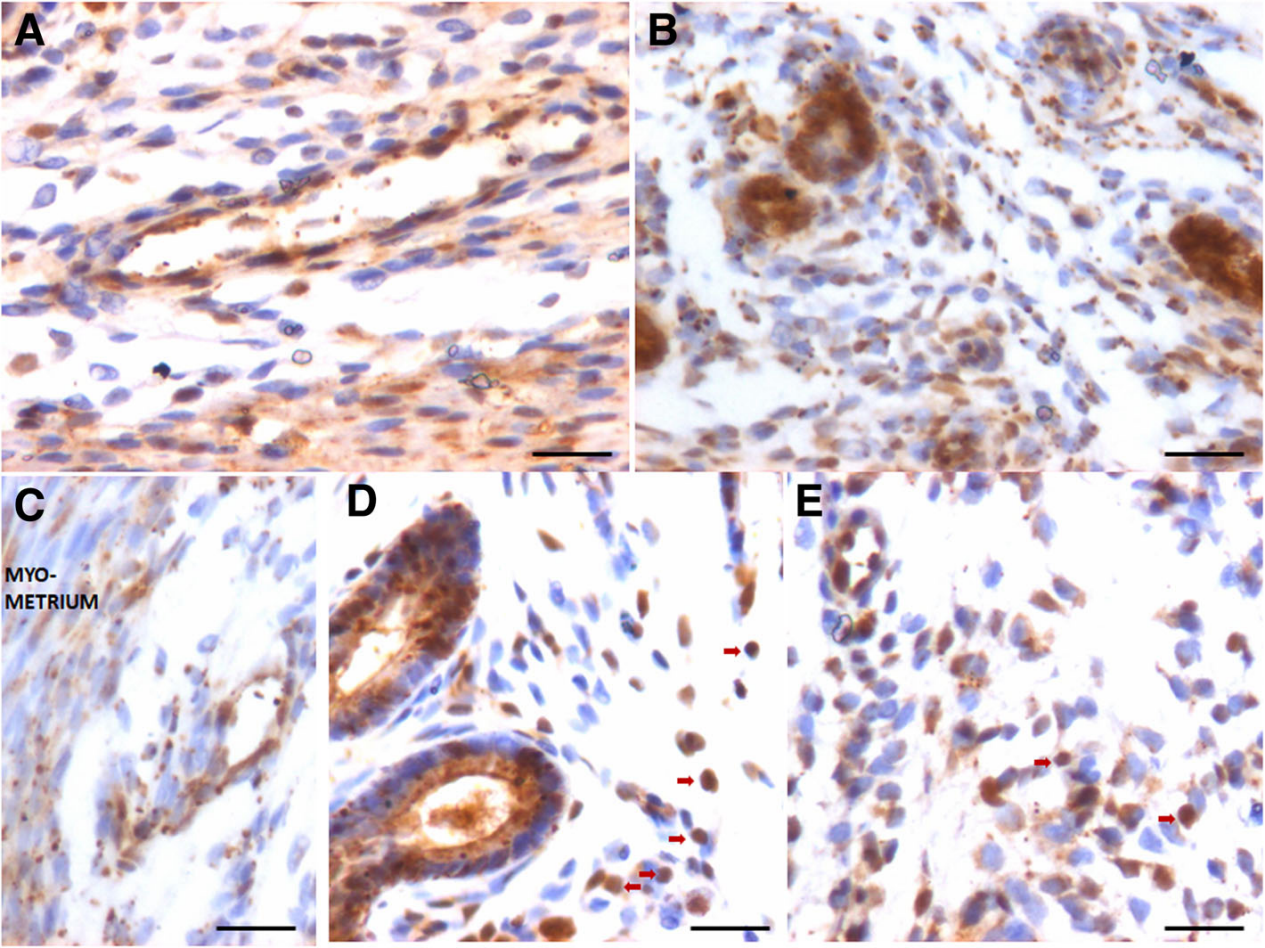
OCT-4 expression in the stromal compartment after estrogen (**a**), progesterone (**b**, **c**) and FSH (**d**, **e**) treatment to bilaterally ovariectomized mice. Small spherical cells with nuclear OCT-4 were observed in the lumen of blood vessels, among the stromal cells and also among the glandular epithelial cells. Note that endothelial cells lining the blood vessels, stromal cells and glandular epithelial cells majorly express cytoplasmic OCT-4. Few bigger sized cells with nuclear OCT-4 are also evident (red arrow). Scale bar 20 μm

OCT-4 positive cells were observed in the atrophied uterine sections and majority of epithelial cells invariably expressed cytoplasmic OCT-4. A careful examination showed few, small sized, spherical cells with nuclear OCT-4 (Fig. 5, lower panel). OCT-4 expression was increased after E (upper panel) & P (lower panel) treatment with majority of epithelial cells showing cytoplasmic OCT-4 (Fig. 8). Interestingly the endothelial cells lining the blood vessels also showed cytoplasmic OCT-4 (Fig. 8d, e). Only a few small, spherical cells expressed nuclear OCT-4 and these cells were present among epithelial cells, stromal cells and in the lumen of blood vessels (Fig. 9d-e). Several OCT-4 positive spherical stem cells (of variable size due to different plane of section) became visible after FSH treatment (Fig. 9) at the basal region of epithelial cells as well as in the glands. A careful examination of stromal compartment showed similar OCT-4 positive spherical cells among the stromal cells and in the lumen of blood vessels. Also the endothelial cells surrounding the blood vessels showed nuclear to cytoplasmic OCT-4. Majority of stromal cells expressed minimal cytoplasmic OCT-4 or were negative for OCT-4. Figure 10 shows small sized, spherical cells with nuclear OCT-4 in the stromal compartment and in the lumen of blood vessels after various treatments. The endothelial cells lining the blood vessels were relatively bigger and elliptical in shape with cytoplasmic OCT-4.

To conclude, using an antibody that readily detects expression of both the alternatively spliced isoforms OCT-4A (nuclear) and OCT-4B (cytoplasmic), we observed small sized cells with nuclear OCT-4 and majority of epithelial cells with cytoplasmic OCT-4. Small sized cells with nuclear OCT-4 were also observed in the stroma and in the lumen of blood vessels. Since epithelial cells, few stromal cells and endothelial cells lining the blood vessels expressed cytoplasmic OCT-4, it is most likely that these cell types arise by the differentiation of nuclear OCT-4 positive VSELs. Thus we speculate that VSELs being pluripotent, differentiate into epithelial cells, stromal cells as well as give rise to endothelial cells. As these cells differentiate further, cytoplasmic OCT-4 gets degraded. We have earlier reported that similar nuclear OCT-4 positive stem cells give rise to spermatogonial stem cells in testis and to ovarian stem cells in the ovary [28].

### Enrichment of uterine stem cells

**Fig. 11:**
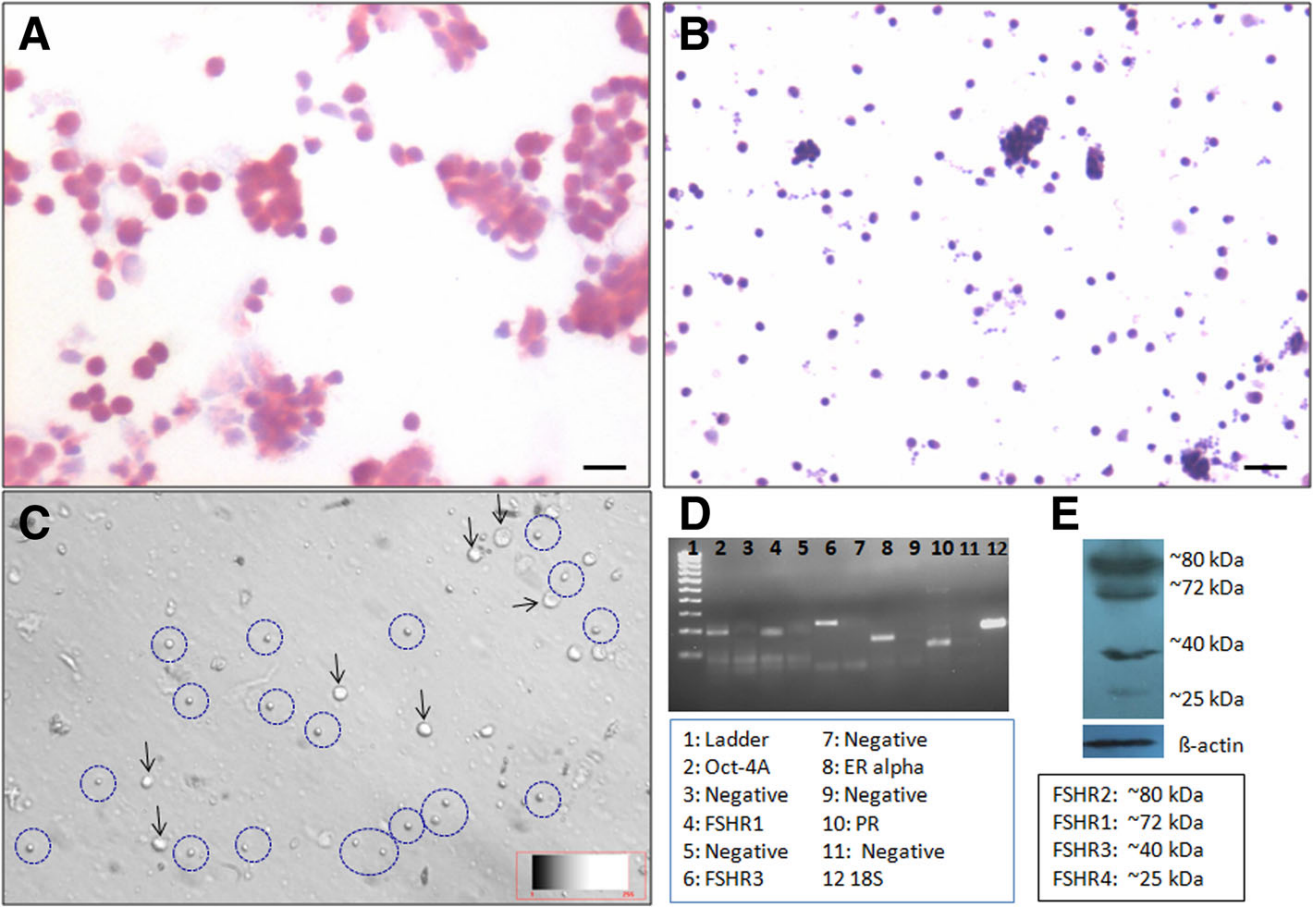
Enrichment of uterine stem cells and detection of ER, PR and FSHR on stem cells. **a** H&E stained uterine cells smears (from progesterone treated mice) obtained after spinning cells at 200 g show presence of cells with abundant pink cytoplasm **b** Small sized, spherical stem cells with high nucleo-cytoplasmic ratio enriched by spinning the supernatant at 1000 g (**c**). The stem cells (broken circle) remain floating after overnight culture along with red blood cells (arrow) whereas the somatic cells get attached to the surface of culture dish. **d** The stem cells thus collected were subjected to RT-PCR analysis. Results show that the cells expressing Oct-4A also express ER, PR as well as both alternatively spliced FSHR isoforms R1 and R3. **e** Western blotting results showed presence of all 4 alternatively spliced FSHR isoforms. Scale bar 20 μm

Uterine stem cells were enriched from progesterone treated mice by using variable speed for centrifugation. Fig. 11 shows cells smears after H&E staining collected after spinning at (A) 1200 and (B) 3000 rpm. Big somatic cells of variable shapes with abundant pink cytoplasm were observed in large numbers after spinning at 1200 rpm. Stem cells collected after spinning at 3000 rpm were very small in size, distinctly spherical in shape, with high nucleo-cytoplasmic ratio and minimal cytoplasm (Fig. 11b). These stem cells remained floating after overnight incubation (Fig. 10c) whereas contaminating somatic cells attached to the surface of the culture dish. The enriched stem cells were used for RNA extraction.

### RT-PCR analysis to study ER, PR and FSHR on the stem cells

ER, PR and FSHR (alternatively spliced Fshr1 and Fshr3) were expressed on enriched population of uterine stem cells (Fig. 11d). Expression of ER and PR is extensively reported on endometrial cells and FSHR was recently reported in human endometrial glandular epithelium by Stilley et al. [45] however, their expression on uterine stem cells is being reported for the first time in the present study. Similar expression of sex hormone and gonadotropin hormone receptors have been reported on ovarian, testicular and hematopoietic stem/progenitor cells [34–37, 44]. Since similar population of pluripotent VSELs exist in various adult tissues, we were not surprised by these results.

### Western blotting to study FSHR isoforms in the uterus

Four bands corresponding to 4 alternately spliced FSHR isoforms were clearly detected in uterine sample (Fig. 11e). The bands observed on Western blotting are similar to earlier resports and were detected using an antibody against the N-terminal region conserved in all the four isoforms of FSHR [40].

### Stem cells proliferation kinetics

**Fig. 12:**
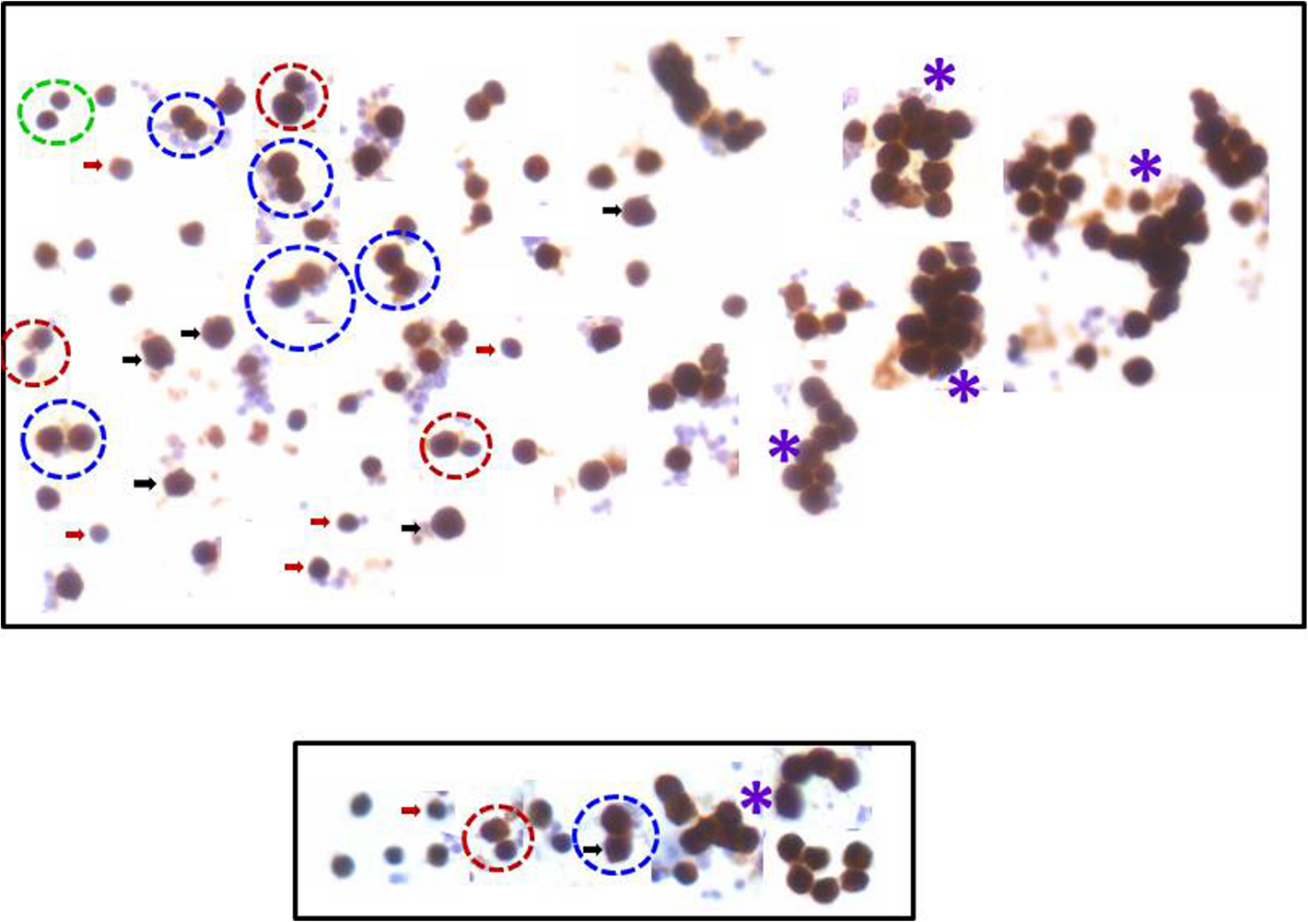
Stem cells proliferation in the uterus. Stem cells were better visualized in smears compared to sections and were successfully enriched by spinning at 3000 rpm (for further details refer to M&M; Fig. 11). These stem cells expressed both PCNA (upper panel) and FSHR (lower panel). This figure is a composite prepared by pasting together several fields. Two distinct stem cells populations based on size were clearly visualized including smaller VSELs (red arrow) and slightly bigger progenitors (black arrow). VSELs are relatively quiescent and undergo self-renewal (green broken circle) and asymmetric cell division (red broken circle) to give rise to the bigger progenitors which undergo symmetric cell divisions (blue broken circle) and clonal expansion (asterix). However, these stem cells in the smears are from what specific region of the uterus is not clear at present since whole uterus was subjected to enzymatic digestion. Similar results have been earlier reported for testis and ovary [37, 41]. Scale bar 20 μm

**Fig. 13:**
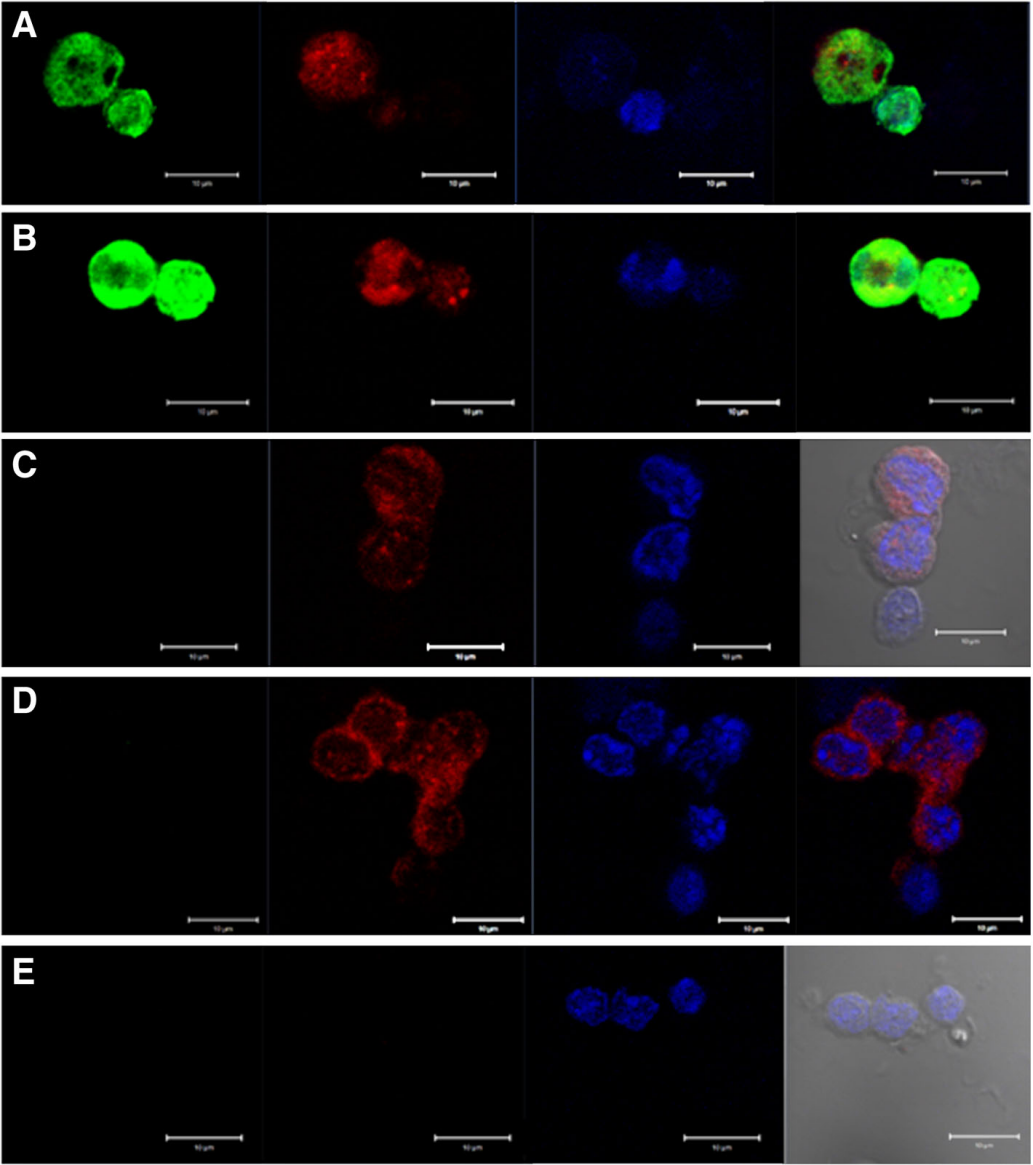
Co-expression of OCT-4 and NUMB in dividing stem cells. OCT-4 was used as a stem cell marker and NUMB as a marker specific for progenitor cells that arise by asymmetric cell division (ACD) from stem cells. **a** ACD was clearly observed wherein the stem cells expressed OCT-4 and NUMB was asymmetrically expressed specifically by the slightly bigger progenitor cell. Stem cells undergoing (**b**, **c**) symmetric divisions and (**d**) clonal expansion expressed NUMB. **e** Negative control with omission of primary antibodies

Distinct cell divisions including asymmetric cell divisions (dividing cells of unequal size, ACD) and symmetric cell divisions (dividing cells of equal size, SCD) and clonal expansion (cell spheres with incomplete cytokinesis) were clearly visualized in cell smears prepared from progesterone treated uterus (Fig. 12). Similar stem cell proliferation kinetics has been reported in other tissues as well [42]. Results suggest that VSELs are the most primitive, pluripotent stem cells in the uterus. Co-expression of OCT-4 (stem cell marker) and NUMB (expressed in progenitor cells) further confirmed that small sized, OCT-4 expressing VSELs undergo ACD to give rise to the progenitors which in turn undergo SCD and clonal expansion (Fig. 13). The results are in agreement with earlier findings of asymmetric distribution of OCT-4 and NUMB in the hematopoietic system [39]. During ACD, NUMB expression was observed restricted only to the bigger progenitor cell (Fig. 13a). The expression pattern was very dynamic and OCT-4 expression was gradually lost as cells differentiated further. Cells undergoing SCD (Fig. 13b-c) and clonal expansion (Fig. 13d) expressed whereas negative control (Fig. 13e) showed no staining.

### Differential expression of various transcripts by qRT-PCR studies

**Fig. 14:**
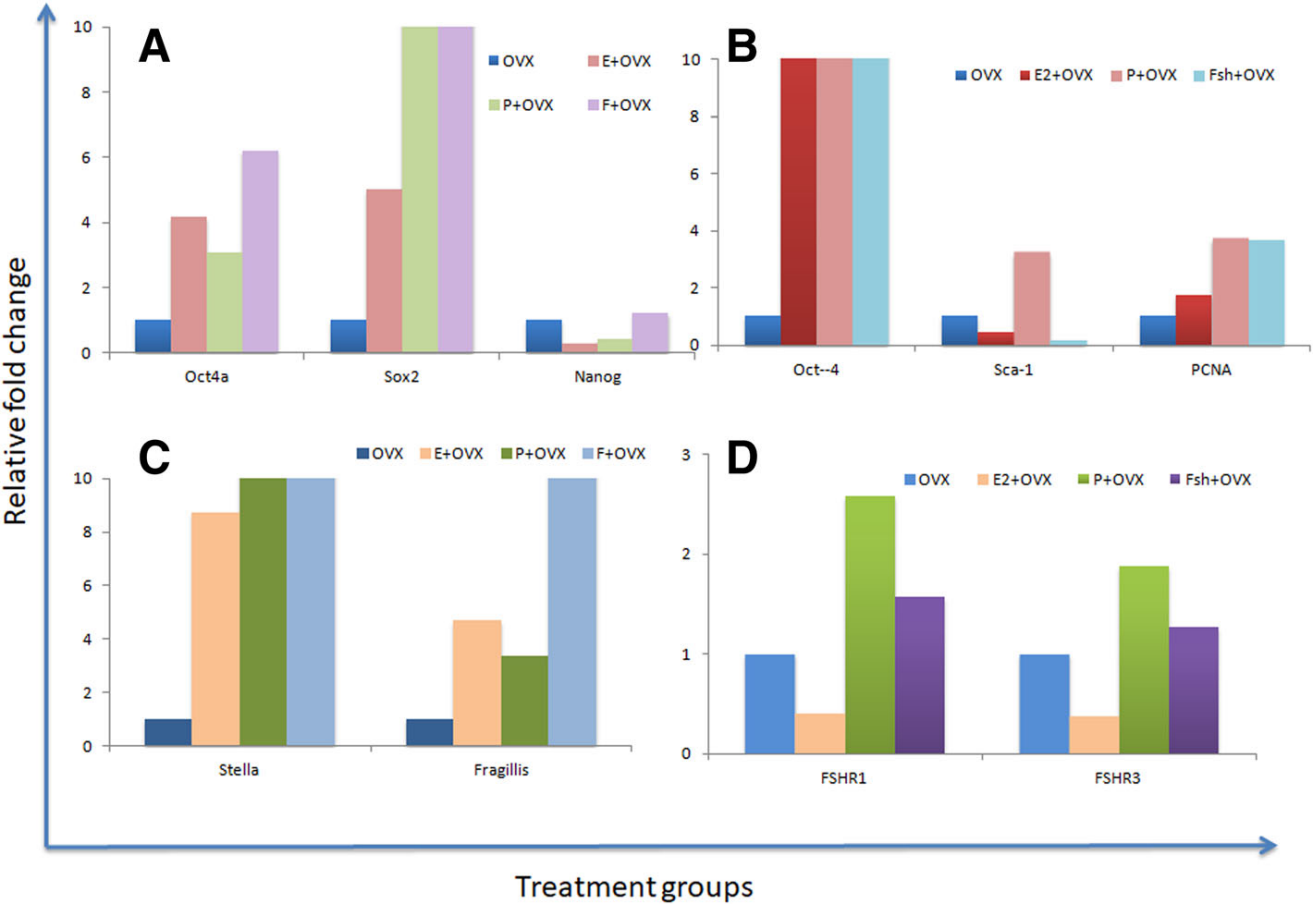
Differential regulation of various transcripts by E,P and FSH treatment to bilaterally ovariectomized mice. qRT-PCR results show up-regulation of (**a**) pluripotent Oct-4A, Sox2 and Nanog (**b**) stem/progenitor (Oct-4, Sca-1) and proliferation (Pcna) (**c**) primordial germ cells (PGCs) Stella, Fragilis (**d**) FSHR isoforms R1 and R3 specific transcripts with respect to ovariectomized uterine samples taken as one. There is an overall increase in various markers as a result of treatment. The study needs to be repeated on more numbers of mice. Scale bar 20 μm

VSELs express both pluripotent and primordial germ cells (PGC) specific markers. A distinct differential expression of pluripotent (Oct-4A, Nanog, Sox2), progenitors (Oct-4, Sca-1), Pcna and primordial germ cells (Stella and Fragilis) specific transcripts was observed after various treatments compared to ovariectomized untreated control. Oct-4A was increased 3–6 folds whereas total Oct-4 was up regulated almost 10 folds. These results suggest that VSELs (with nuclear OCT-4A) were activated, underwent self-renewal and also gave rise to the progenitors (with cytoplasmic OCT-4B) which divided rapidly and underwent clonal expansion. Both the spliced isoforms of FSHR including canonical Fshr1 and growth factor type 1 isoform Fshr3 were found to be up regulated after treatment with P and FSH. (Fig. 14).

### Flow cytometry estimation of VSELs

**Fig. 15:**
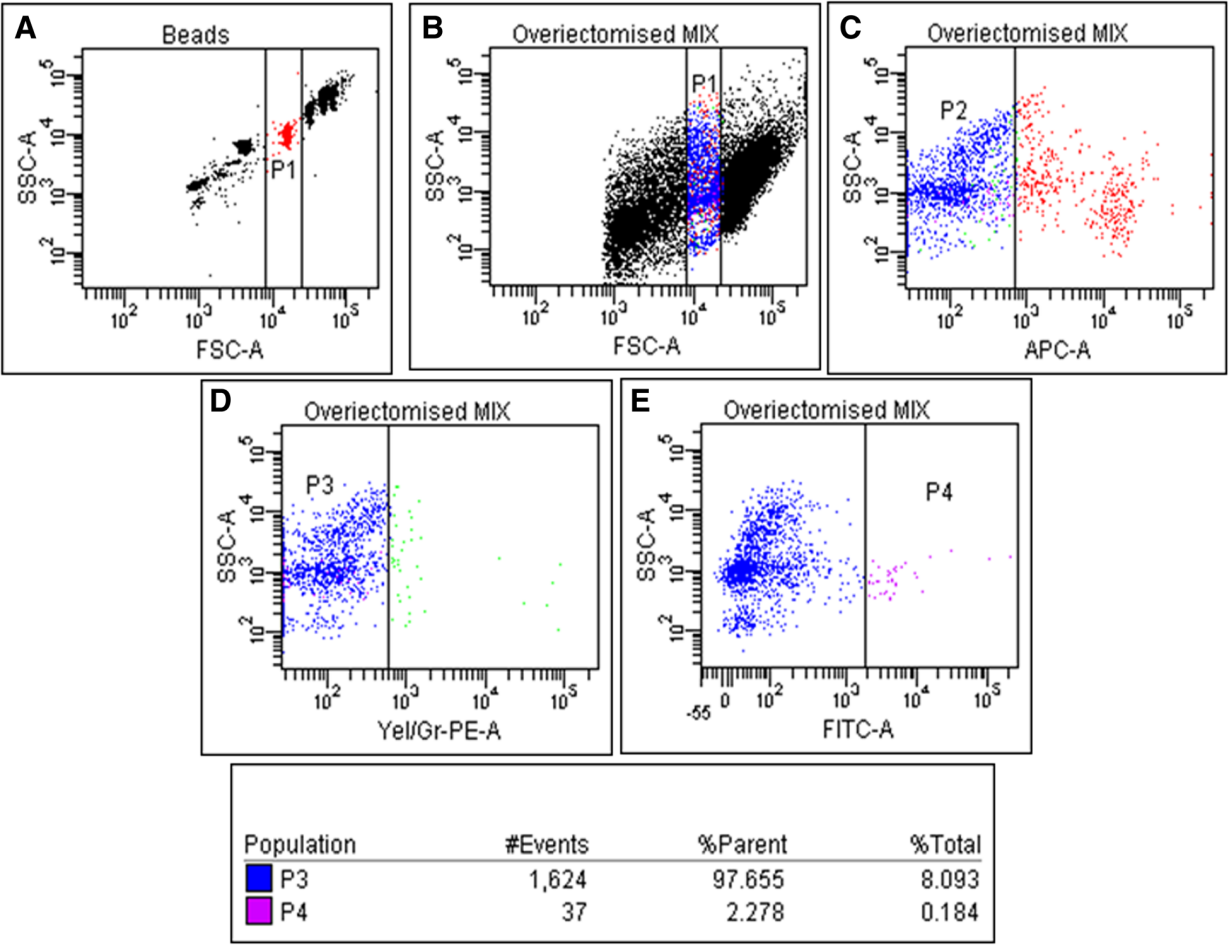
Estimating VSELs in mouse uterine cells by flow cytometry. VSELs with a surface phenotype Lin-/CD45-/Sca-1+ in mouse uterus were studied as described earlier [10] (**b**) cells were gated in the size range of 3–5 μm representing P1 population based on (**a**) calibration beads (**c**) LINEAGE negative cells were further gated as P2 population (**d**) From P2 population, CD45 negative cells (yellow PE tagged) were separated as P3 (**e**) In the P3 population, SCA-1 expressing cells (FITC tagged) were gated further as P4. Based on this gating strategy almost 0.18% events were VSELs in ovariectomized mice. Using similar method we detected 0.56, 1.80 and 1.80% VSELs after E, P & FSH treatment respectively

VSELs were studied using a similar gating strategy (Fig. 15) as described earlier by Ratajczak’s group [43] and later by our group in the mouse uterus [10]. VSELs with a cell surface phenotype of LIN-/CD45-/SCA-1+ were detected in ovariectomized uterus (0.18%) and their numbers increased after treatment with estrogen (0.56%), progesterone and FSH (1.80%).

## Discussion

Results of the present study provide strong evidence in support of pluripotent OCT-4A expressing very small embryonic-like stem cells (VSELs) in the endometrial compartment of adult mouse uterus similar to their presence recently reported in the myometrium and perimetrium [11] and in agreement with our earlier pilot study [10]. Stem cells were found located among the epithelial cells lining the lumen and glands, stromal compartment as well as in the lumen of blood vessels. Stem cells expressed transcripts specific for pluripotent state (Oct-4A, Sox2, Nanog), primordial germ cells (Stella, Fragilis), progenitors (Oct-4, Sca-1) and receptors for ER, PR and FSHR. All these markers along with Pcna (proliferation marker) were up-regulated to varying extent in response to treatment. Alternately spliced FSHR isoforms were detected in the uterine tissue. Flow cytometry analysis detected small, 2–5 um VSELs with a LIN−/ CD45−/ SCA-1+ surface phenotype that underwent self-renewal, asymmetric and symmetric cell divisions along with clonal expansion with incomplete cytokinesis. Results suggest that nuclear OCT-4 positive VSELs possibly undergo asymmetric cell divisions and are the most primitive stem cells in the uterus that give rise to progenitors that further differentiate into epithelial cells, stromal and endothelial cells (all expressed cytoplasmic OCT-4). These results are in agreement with our earlier findings in testis [37] and ovary [46] and provide additional information to the current understanding of stem cells biology in the uterus, their regulation by sex and gonadotropin hormones and provide further insight in addition to the 10 years progress on uterine stem cells reviewed by Gargett et al. [1]. The stem cells became better detectable in response to treatment with higher dose of hormonal treatment. Under normal conditions they perform their activity in a very subtle manner and have eluded the scientific community till now. We also did not detect them in histological sections in our earlier report where physiological doses of steroids were used for treatment [10]. We have earlier reported that FSH acts on stem cells via alternatively spliced Fshr3 in ovaries and testis [34, 37, 46]. Thus it will be ideal to study FSHR expression on a pure population of uterine stem cells at early time points (3 and 24 h) after FSH treatment.

The underlying reason why mesenchymal cells have been extensively reported as possible endometrial stem cells by various investigators [1, 2] is because of their abundance and ease to multiply in vitro. Early passages of MSCs harbor a sub-population of pluripotent VSELs [9] which do not divide rapidly in culture as they are relatively quiescent and thus MSCs become the pre-dominant cell type in later passages. It has recently been reported that MSCs are indeed pericytes that expand easily in culture and can be isolated from any tissue source [5, 6]. The whole confusion related to the true identity of uterine stem cells has occurred because it is easier to study stem cells in culture rather than in primary tissue and secondly because of rare occurrence and small size of VSELs. This makes results of the present study performed on tissue sections unique and novel.

Gargett’s group [47] attempted to detect uterine stem cells involved in regeneration using mTert-GFP transgenic mice (with green fluorescent protein GFP reporter under the control of the telomerase reverse transcriptase promoter). mTert is part of telomerase complex that is expected to be present in stem cells to maintain telomere length. However, no clear results emerged from their study and it was concluded that telomerase activity reflects leucocyte population rather than stem cells. These conclusions were surprising and suggest that true stem cells eluded their study because of their small size and rare occurrence. In contrast to their lineage tracing model, we used a unique strategy to isolate the stem cells based on their very small size and our knowledge regarding their inability to pellet down at normal speed of 200–250 g [28]. Expression of pluripotent and PGC specific markers confirm their pluripotent state and source of origin. Similar stem cells exist in all adult organs in the body and the work was recently reviewed [27, 28].

Based on OCT-4 expression pattern wherein nuclear OCT-4A reflects pluripotent state and cytoplasmic OCT-4B is suggestive of differentiated/committed state; we propose that the pluripotent, nuclear OCT-4A positive VSELs in the uterus differentiate into cytoplasmic OCT-4B expressing epithelial, stromal and endothelial cells. In a parallel study, we have gathered evidence to suggest that nuclear OCT-4 positive VSELs located in the perimetrium differentiate and give rise to the myometrial and endothelial cells expressing cytoplasmic OCT-4B [11]. Similar nuclear and cytoplasmic OCT-4 localization (reflecting spliced variants OCT-4A and OCT-4B) in pluripotent and non-pluripotent human PGCs has been reported [38]. It was proposed that OCT-4A in PGCs either translocates to the cytoplasm or is attenuated there possibly for degradation as the significance of cytoplasmic OCT-4 is otherwise unknown, and is notably coincident with major global epigenetic changes.

A true stem cell is expected to undergo rare, asymmetric cell divisions (ACD) [41, 48, 49] whereas progenitors divide rapidly by symmetric cell divisions (SCD) and undergo clonal expansion and this is also expected from uterine stem cells VSELs are possibly the most primitive stem cells in the uterus that undergo ACD to give rise to the progenitors which undergo SCD and clonal expansion before further differentiation into various cell types [42]. Similar to ovary and testis, in the uterus also two distinct populations of stem cells (based on size) were detected which underwent ACD, SCD and clonal expansion. Based on the presence of many more stem cells after FSH treatment (Fig. 4) compared to significant hyperplasia after P treatment (Fig. 3), we could speculate that whereas FSH induces self-renewal of stem cells, P is required for their further differentiation into progenitor cells and E results in their growth and hypertrophy. Further in vitro studies need to be undertaken to decipher the role of various hormones on stem cells biology in the uterus.

A crucial role of the stem cells niche emerges which decides the stem cell fate based on their location. The same VSELs in the hematopoietic system differentiate into blood cells, in the testis give rise to sperm, in ovary give rise to oocytes and in the uterus differentiate into epithelial, stromal, endothelial as well as myometrial cells. The niche possibly controls specific epigenetic changes that occur in the pluripotent stem cells with open euchromatin as they enter differentiation into tissue specific ‘progenitors’ (only specific chromatin remains active, rest gets inactivated) thus controlling their final fate. We have recently shown that mouse bone marrow VSELs when placed on a bed of Sertoli cells (that acts as the somatic niche) can differentiate into germ cells [32].

True to their quiescent nature, VSELs survive in atrophied uterus as shown in the present study in agreement with earlier pilot study [10] and also in chemoablated gonads and bone marrow [28]. They serve as a backup pool of stem cells and give rise to uterine progenitors throughout life. VSELs are activated in response to any kind of stress, get mobilized and undergo self-renewal to restore homeostasis. Thus we are tempted to propose that activation of pluripotent stem cells may be the underlying mechanism how endometrium regenerates every cycle and prepares itself for implantation. Gunjal et al. [10] using physiological doses of E & P for treatment, after careful and prior sensitization of bilaterally ovariectomized mouse with E, showed that maximal transcripts for Oct-4A and Oct-4 exist during receptive phase (E + P) and during endometrial regeneration/remodeling (48 h withdrawal of E + P). Results of the present study need to be confirmed by others in the field and provide huge scope for further research.

For the sake of clarity and to explain and discuss the relevance of our results, further discussion is broken down into sub-headings.

### Epithelial cells proliferation in response to steroid hormones

It is a widely accepted fact that proliferation of epithelial cells is mediated indirectly via growth factors secreted by the stromal cells in response to estrogen treatment. Besides Igf-1, Hgf, Tgfα, Fgf reported earlier by various investigators, recently Fgf10 and Bmp8 secreted by stromal cells have been demonstrated to mediate estrogen action on epithelial proliferation [50] via ERα. Tissue recombination studies (where one cell type lacked ER), between epithelium and stroma and later transplantation on kidney provided indirect evidence that stroma may exert a paracrine influence on epithelial cells. However, direct evidence to support this concept is scarce. This concept fails to also explain aberrant proliferation of epithelial cells and role of estrogen in epithelial cancers and endometriosis. PR are understood to be induced by estrogen via ER and P antagonizes E-mediated cell proliferation and induces differentiation in the receptive phase. But if this was true and P does not induce hyperplasia, why are PR also expressed on endometrial cancer cells? Present study provides interesting perspective to mechanism of action of steroid hormones E & P on uterine stem cells. *Rather than an indirect action of estrogen* via *stromal cells resulting in epithelial cells proliferation, it is possibly the VSELs (that express ER/PR/FSHR) located amongst the epithelial cells that respond to ovarian  hormones and FSH directly by undergoing self-renewal/ACD/SCD and clonal expansion and give rise to the progenitors which further differentiate into epithelial cells with cytoplasmic OCT-4.*

It is also intriguing to note that whereas high dose of E resulted in hypertrophy (tall cells with more pink stained cytoplasm) of epithelial cells, high dose of P resulted in conspicuous overcrowding of blue stained epithelial cells nuclei (rapid nuclear divisions and hyperplasia) with higher PCNA expression. This implies that stem cells are more activated by P compared to E treatment. Published literature suggests a pivotal role of P in endometriosis as well as fibroids [51, 52]. Both endometriotic lesions and eutopic endometrium show sustained proliferation even in the P dominated secretory phase. Rather than interpreting these results as sustained proliferation due to P resistance, results of present study suggest that sustained proliferation in P dominated secretory phase could be a direct effect of P on stem cells resulting in hyperplasia of stem/progenitors in fibroids as well as endometriosis. This was recently discussed [53]. Our earlier study [10] showed higher expression of OCT-4 (reflecting increased numbers of progenitors) in P treated group. Higher dose of treatment in the present study showed increased numbers of stem cells in P treated mice compared to E treated group. These results challenge existing understanding of hormone action on the endometrial cells and need to be better understood.

### Extra-gonadal action of FSH on mouse endometrium

Surprisingly, FSH treatment to ovariectomized mice resulted in increased numbers of stem cells and hypertrophy of epithelial cells which were easily visualized in H&E stained sections and supported by RT-PCR and qRT-PCR results. Four alternately spliced FSHR isoforms detected by Western blotting, using an antibody against the N-terminal region of FSHR (conserved in all the isoforms) were similar to the reported four isoforms of FSHR [39]. Two of the isoforms Fshr1 and Fshr3 transcripts were also detected by qRT-PCR. Our findings suggest that FSH possibly exerts a direct action on the uterine stem cells. These results are novel and challenge existing understanding in the field that FSH acts exclusively on the gonads and FSHR are expressed exclusively on granulosa cells in ovary and on Sertoli cells in testes. Few published reports provide further support to our findings. La Marca et al. [54] detected FSHR expression on human endometrium which was up regulated in the secretory phase of the menstrual cycle suggesting FSH role in the regulation of endometrial function and embryo-endometrium interaction. Stilley et al. [45] also reported FSHR on placenta, uterine tissue during pregnancy, non-pregnant endometrium in both proliferative and secretory phases, cervical glandular epithelium, muscle fibers, non-pregnant myometrium, cervix, endothelial cells and arterial smooth muscle cells. Kumar [55] in his commentary on Stilley’s work commented that there is an urgent need to understand the underlying mechanism and function of FSHR in extra-ovarian sites. Ubiquitous expression of FSHR on various adult organs and tumor tissues possibly reflects novel VSELs biology [44].

Work done in our laboratory and by others has shown FSHR expression on stem cells/progenitors in testis [37], ovary [34, 46], cord blood [32] and bone marrow [35, 36, 56]. Sex hormones and FSHR were also reported on hematopoietic stem cells [57]. Present study reveals similar FSHR expression on the stem cells in the uterus also. VSELs are pluripotent stem cells that express sex hormone and FSH receptors (irrespective of their location) and their differentiated progenies continue expressing FSHR. We believe that the primary role of FSH is to stimulate stem cells to undergo self-renewal and further ACD/SCD/clonal expansion and FSHR expression on differentiated cells possibly represents a redundant protein which will eventually be degraded.

### Oct-4 expression in endometrium, endometriosis and endometrial cancer

Several groups have reported OCT-4 in endometrium and various associated pathologies but none of the prior studies appreciated alternatively spliced isoforms and majority reported cytoplasmic OCT-4 expression as shown in Table 2. Pachiarotti et al. [16] for the first time reported nuclear OCT-4 in endometriotic tissue and later Gunjal et al. [10] for the first time showed the presence of very small embryonic-like stem cells (VSELs) with nuclear OCT-4 along with slightly bigger cells with cytoplasmic OCT-4 and their regulation by hormones. Nuclear versus cytoplasmic OCT-4 expression reported by various groups depends on the choice of antibody used for the study. Expression of nuclear OCT-4 lends credence to the possibility that it is the uncontrolled proliferation of nuclear OCT-4A positive VSELs that result in various pathological conditions like endometriosis and cancer. Possible involvement of VSELs in initiating cancers was first proposed by Ratajczak’s group [58] and recently reviewed by us [59]. *Results of the present study provide evidence for the endometrial stem cells/progenitor cells serving as the ‘seeds’ for endometriosis as well as cancer.*

**Table 2:** List of publications reported OCT-4 expression in endometrial samples

Reference	Study results in brief
Matthai et al., 2006 [13]	All 9 of 9 samples studied showed OCT-4 by RT-PCR and protein was expressed in the cytoplasm of few stromal cells.
Forte et al., 2009 [14]	Differential expression of stemness markers (SOX2, SOX15, ERAS, SALL4, OCT4, NANOG, UTF1, DPPA2, BMI1, GDF3, ZFP42, KLF4, TCL1) were studied in endometrial and endometriotic tissue by RT-PCR. OCT-4 was detected in all the samples studied
Bentz et al., 2010 [15]	OCT-4 expression was studied in human endometrial samples in both follicular and luteal phase. They detected mRNA in all samples (49 follicular and 40 luteal phase samples) and concluded that OCT-4 expression restricted to the cytoplasm and was not modulated by hormones.
Pachiarotti et al., 2011 [16]	Reported nuclear OCT-4 expression in epithelial and stromal cells of both eutopic and endometriotic endometrium for the first time. They reported 10 fold higher and more intense nuclear OCT-4 expression in ectopic endometriotic tissue both in ovarian and peritoneal lesions. The group concluded that endometriosis has a stem cell origin and could lead to ovarian cancer.
Zhou et al., 2011 [17]	Detected expression of Oct-4, Sox2 and Nanog in endometrial adenocarcinoma samples
Chang et al., 2013 [18]	Transcription of OCT4 gene was found significantly up-regulated in human ectopic endometriotic tissues. They concluded that expression of OCT4 may contribute to the pathology of ectopic endometrial growth by stimulating the migration activity of endometrial cells.
Song et al., 2014 [19]	Studied normal women and those with ovarian endometriosis. Sox2, Nanog and Oct-4 expression studied by qRT-PCR, Western blotting and IHC. Although they observed higher expression of Nanog and Sox2, they found a trend towards lower OCT4 mRNA and higher OCT4 protein expression in ectopic endometrium.
Pitynski et al., 2015 [20]	Co-expression of SOX-2 and Oct4 by IHC and their correlation with clinic-pathological features of endometrial adenocarcinomas (EACs) was investigated. Reported nuclear OCT-4 and SOX2 in endometrial adenocarcinoma tissue.
Gunjal et al., 2015 [10]	Reported pluripotent VSELs expressing nuclear OCT-4 in adult mouse endometrium for the first time. Reported regulation of stem cell markers (Oct-4A, Oct-4, Nanog, Sca-1) by circulating hormones
Davoudi et al. 2016 [21]	mRNA expression of Oct4 and Sox2 in the uterine tissues of ovariectomized mice was regulated by hormones
Proestling et al., 2016 [22]	Reported co-localization of SOX15 and OCT4 in epithelial and stromal cells of endometriotic tissue. Results support the hypothesis that up-regulation of stem cell-related factors contribute to the establishment of endometriotic lesions.

To conclude, present study has provided further novel insights into stem cells biology in the endometrium and will prove to be a game changer as it challenges existing concepts regarding hormone action, how endometrium undergoes regeneration, FSH action on endometrium and also identified stem cells that could possibly initiate pathologies like endometriosis and cancer.

## Abbreviations

E: Estradiol
FSH: Follicle stimulating hormone
MSCs: Mesenchymal stromal/stem cells
P: Progesterone
PGCs: Primordial germ cells
VSELs: Very small embryonic-like stem cells
