# Correction to: Gonadotropin and steroid hormones regulate pluripotent very small embryonic-like stem cells in adult mouse uterine endometrium

**DOI:** 10.1186/s13048-023-01146-w

**Published:** 2023-04-11

**Authors:** Kreema James, Deepa Bhartiya, Ranita Ganguly, Ankita Kaushik, Kavita Gala, Pushpa Singh, S. M. Metkari

**Affiliations:** Stem Cell Biology Department, ICMR - National Institute for Research in Reproductive Health, Jehangir Merwanji Street, Parel, Mumbai, 400 012 India

**Correction: J Ovarian Res 11:3 (2018)**

10.1186/s13048-018-0454-4

Following publication of the original article [1], the authors identified that the image of Fig. 10a was incorrect. The correct Fig. 10 is shown in the following page of this article.

**Fig. 10:**
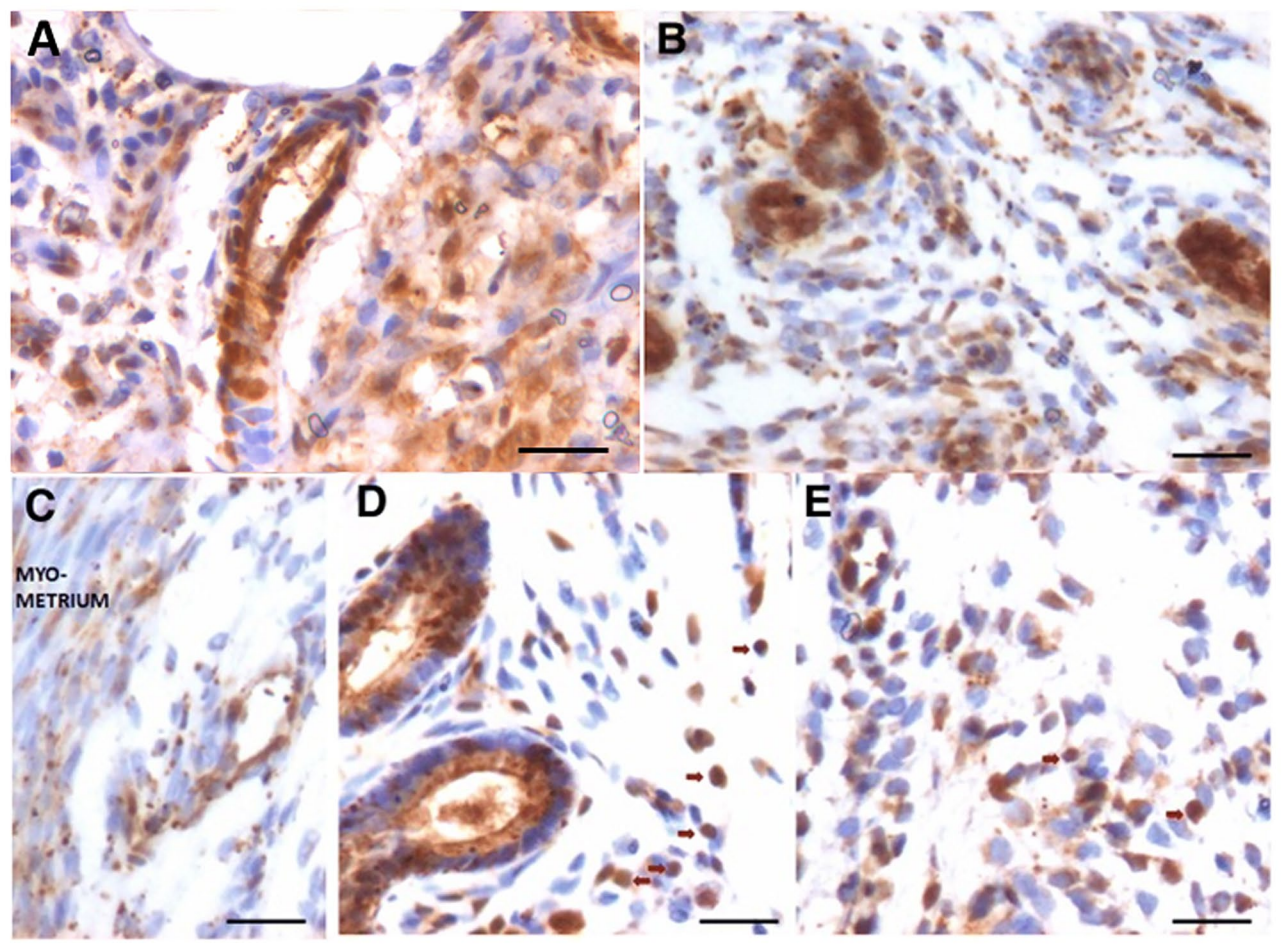
OCT-4 expression in the stromal compartment after estrogen (**a**), progesterone (**b**, **c**) and FSH (**d**, **e**) treatment to bilaterally ovariectomized mice. Small spherical cells with nuclear OCT-4 were observed in the lumen of blood vessels, among the stromal cells and also among the glandular epithelial cells. Note that endothelial cells lining the blood vessels, stromal cells and glandular epithelial cells majorly express cytoplasmic OCT-4. Few bigger sized cells with nuclear OCT-4 are also evident (red arrow). Scale bar 20 μm
